# Intraspecific Variation within the *Utricularia amethystina* Species Morphotypes Based on Chloroplast Genomes

**DOI:** 10.3390/ijms20246130

**Published:** 2019-12-05

**Authors:** Saura R. Silva, Daniel G. Pinheiro, Helen A. Penha, Bartosz J. Płachno, Todd P. Michael, Elliott J. Meer, Vitor F. O. Miranda, Alessandro M. Varani

**Affiliations:** 1 Departamento de Tecnologia, Faculdade de Ciências Agrárias e Veterinárias, Jaboticabal, Universidade Estadual Paulista (Unesp), Sao Paulo 14884-900, Brazil; daniel.pinheiro@unesp.br (D.G.P.); helen.penha@gmail.com (H.A.P.); 2 Department of Plant Cytology and Embryology, Institute of Botany, Faculty of Biology, Jagiellonian University in Kraków, 30-387 Krakow, Poland; bartosz.plachno@uj.edu.pl; 3 J. Craig Venter Institute, La Jolla, CA 92037, USA; tmichael@jcvi.org; 4 10X Genomics, Pleasanton, CA 94566, USA; ejmeer@gmail.com; 5 Departamento de Biologia Aplicada à Agropecuária, Faculdade de Ciências Agrárias e Veterinárias, Jaboticabal, Universidade Estadual Paulista (Unesp), Sao Paulo 14884-900, Brazil

**Keywords:** *Utricularia amethystina*, Lentibulariaceae, chloroplast phylogenomics, organelle genome, carnivorous plants, polymorphic species, intraspecific variation, *ndh* genes

## Abstract

*Utricularia amethystina* Salzm. ex A.St.-Hil. & Girard (Lentibulariacea*e*) is a highly polymorphic carnivorous plant taxonomically rearranged many times throughout history. Herein, the complete chloroplast genomes (cpDNA) of three *U. amethystina* morphotypes: purple-, white-, and yellow-flowered, were sequenced, compared, and putative markers for systematic, populations, and evolutionary studies were uncovered. In addition, RNA-Seq and RNA-editing analysis were employed for functional cpDNA evaluation. The cpDNA of three *U. amethystina* morphotypes exhibits typical quadripartite structure. Fine-grained sequence comparison revealed a high degree of intraspecific genetic variability in all morphotypes, including an exclusive inversion in the *psb*M and *pet*N genes in *U. amethystina* yellow. Phylogenetic analyses indicate that *U. amethystina* morphotypes are monophyletic. Furthermore, in contrast to the terrestrial *Utricularia reniformis* cpDNA, the *U. amethystina* morphotypes retain all the plastid NAD(P)H-dehydrogenase (*ndh*) complex genes. This observation supports the hypothesis that the *ndh*s in terrestrial *Utricularia* were independently lost and regained, also suggesting that different habitats (aquatic and terrestrial) are not related to the absence of *Utricularia* *ndh*s gene repertoire as previously assumed. Moreover, RNA-Seq analyses recovered similar patterns, including nonsynonymous RNA-editing sites (e.g., *rps14* and *petB*). Collectively, our results bring new insights into the chloroplast genome architecture and evolution of the photosynthesis machinery in the Lentibulariaceae.

## 1. Introduction

The species of the carnivorous plant family Lentibulariaceae are grouped in three genera: *Pinguicula* L., *Genlisea* A.St-Hil., and *Utricularia* L. [1,2], and are increasingly becoming important plant models mainly due to their alternative nutrient uptake system, their morphological non-orthodox body structure, characterized by Fuzzy Arberian Morphology [3,4], and particular genomic characteristics, such as high mutational levels with nuclear genome shrinkage and expansion in some lineages [5,6].

*Utricularia* is the biggest genus and most widespread group of carnivorous plants and is very diverse regarding its distribution and habit (e.g., terrestrial, aquatic, lithophytes, epiphytes, and reophytes) [7]. Moreover, several species are polymorphic, which may lead to controversial taxonomic classification. For instance, *Utricularia amethystina* Salzm. ex A.St.-Hil. & Girard is a terrestrial herb, with petiolate and rosetted leaves. The species is broadly distributed in about 18 different countries of the tropical and subtropical America [7], commonly found in different altitudes (from inselbergs in the Guianas [8] to the coast of Brazil [9]) and habitats such as humid sandy soil of the savannas, swamps, and soil between rocks usually near streams, rivers, and waterfalls.

**Figure 1:**
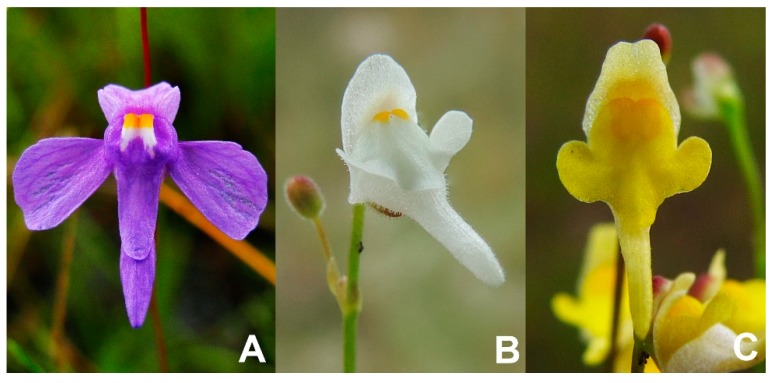
*Utricularia amethystina* species morphotypes are differentiated mainly by corolla color. (**A**) *U. amethystina* purple morphotype; (**B**) *U. amethystina* white morphotype; (**C**) *U. amethystina* yellow morphotype.

The species is classified in the *Utricularia* sect. *Foliosa* Kamieński, showing common morphological characteristics making it easy to distinguish from other *Utricularia* sections due to its connate bracts and bracteoles, which is the singular morphology of utricles (carnivorous traps), and the capsule dorsoventrally and bivalvate dehiscence. However, the distinction between the conspecific species is not a trivial task, as *U. amethystina* shows high intraspecific variation, mainly between reproductive characters, such as the corolla shapes and colors, which can vary from shades of purple (Figure 1A), white (Figure 1B) to yellow (Figure 1C) [7].

This intraspecific morphological variation resulted in several taxonomic rearrangements since the earliest descriptions at nineteenth century [10,11] and even now there is much controversy about if the species is one or more [12,13]. Taylor (1989) [7] struggled to separate the species based on reproductive characters, such as corolla shape, pedicel sizes, palynological characters, and calyx indumentum, but he couldn’t find traits for enough taxonomical circumscriptions to split the different morphotypes. Indeed, in his *Utricularia* taxonomic monograph, he synonymized 31 taxa under the binomial “*Utricularia amethystina*” and he wrote “*U. amethystina* is a most ‘difficult’ and excessively polymorphic species...” (Taylor, 1989 [7], p. 291). Therefore, he assumed one name for the species, as he was unable to find discontinuities to support taxa separation due to the high degree of polymorphism between populations. However, to date, there is no proper taxonomic treatment to solve this question. In addition, only a few genetic differences have been explored [13], such as the chloroplast regions *rps*16, *trn*L-F, *trn*D-T, and nuclear ITS, but these markers were not able to give enough resolution to distinguish them all. In this context, chloroplast genomes are a valuable resource for phylogenies, and the study of their structure and content can provide clues for improving inter- and intraspecific studies, such as population biology [14], and even the discovery of new species [15].

The chloroplast genomes of most angiosperms have conserved quadripartite structure separated in Large and Small Single Copy regions (LSC and SSC, respectively) and two inverted repetitive regions (IRs) [16]. However, comparative analyses indicate that some plants, such as parasitic [17], mycoheterotrophic (e.g., in [18,19]), and species of carnivorous plants from the order Caryophyllales [20,21], have suffered substantial rearrangement and gene losses throughout plant evolution. For example, across diverse lineages of plants, chloroplast genomes lack NAD(P)H-dehydrogenase (*ndh*) complex genes, genes that could have been involved in the transition from aquatic to terrestrial habit thought plant evolutionary history [22,23].

Within Lentibulariaceae family, there are published chloroplast genomes (cpDNA) of *Pinguicula ehlersiae* [24] and seven *Genlisea* species [24,25]. There are cpDNA genomes available for four *Utricularia* species: *U. gibba* [26], *U. macrorhiza* [24], *U. reniformis* [27], and *U. foliosa* [28].

All published *Utricularia* cpDNAs have the typical quadripartite structure and the same genes as most angiosperms. However, some species exhibit variation, such as two complete copies of *ycf*1 and *ndh*F in *U. gibba*, and *U. reniformis*, which has reduced chloroplast size due to several losses in all *ndh*s genes repertoire that could not be integrally found either in the mitochondrial genome [29] nor in the nuclear DNA (unpublished data). Indeed, *U. reniformis* is a terrestrial species and other assessed *Utricularia* are aquatic, and based on this observation, Silva et al. (2016) proposed that the loss of *ndh*s could be related to terrestrial habit [27]. Nonetheless, other studies are still needed for a better understanding of genes especially the evolution of the *ndh* genes in the genus.

Herein, we present the chloroplast genomes of three *Utricularia amethystina* morphotypes to assess inter- and intraspecific sequence variability and polymorphic regions that could be used for further phylogenetic studies. In addition, we employed chloroplast transcriptome to assess gene expression and identify the RNA editing sites in each chloroplast. We also compare *ndh* gene gains and losses across the sequenced *Utricularia* cpDNA species, to examine the variation of structural changes across the genus.

## 2. Results

### 2.1. Structure of Chloroplasts in Utricularia amethystina

**Figure 2:**
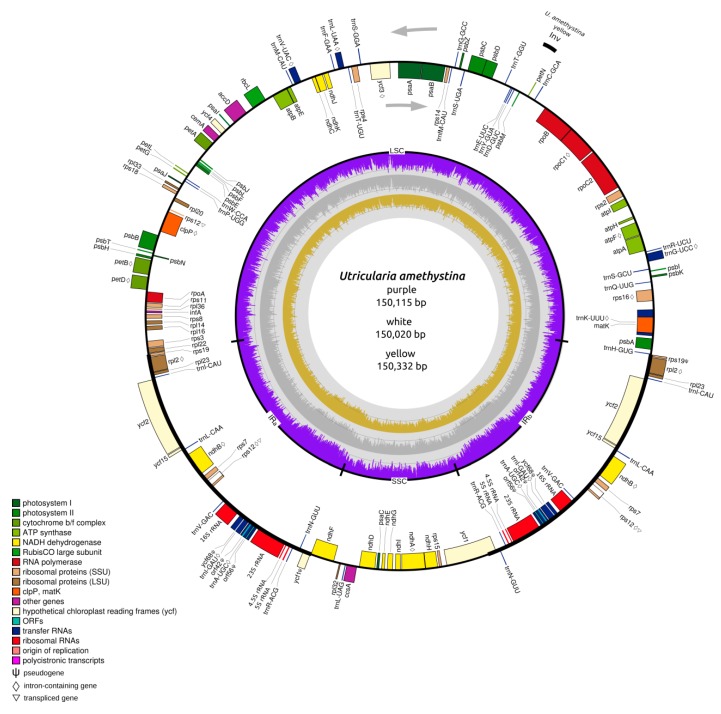
Chloroplast genome map for *Utricularia amethystina* purple, white, and yellow. The map represents all three cpDNAs. Gene order and number are the same, except that yellow has an inversion in the *pet*N and *psb*M genes (see at 2 o’clock in the map). Black thick lines of the outer circle indicate the extension of the inverted repeats. The direction of the arrows denotes the transcription direction. Genes are colored according to their functional groups. The inner graph corresponds to the GC content for each cpDNA region in the chloroplast of each species morphotype. Purple, yellow, and gray bars denote *U. amethystina* purple, yellow, and white morphotypes, respectively.

A total of 2,873,574 million paired-end reads were generated of all *Utricularia amethystina* morphotypes. Approximately 7.57%, 9.10%, and 9.56% represents cpDNA-derived reads and were used for the de novo assembly of *U. amethystina* purple, white, and yellow, respectively. For each morphotype, the assembly using SPAdes resulted in a contig with the entire LSC region, followed by two contigs containing the IR and SSC regions. The cpDNA contigs were joined in a supercontig and circularized using MITOBim iterative read mapping. The three *U. amethystina* cpDNAs have a consistent quadripartite structure similar to the majority of other angiosperms, and slightly varying in size (Figure 2) (Table 1) (Supplementary Table S1).

The three cpDNA exhibited in total of 137 annotated genes, including 39 unique protein-coding genes, 30 tRNA, and four rRNA. Eighteen genes (*pet*B, *pet*D, *atp*F, *rpo*C1, *rps*12, *rps*16, *rpl*2, *ndh*A, *ndh*B (2×), *trn*K-UUU, *trn*A-UGC (2×), *trn*I-GAC (2×), *trn*G-UCC, *trn*L-UAA, *trn*V-UAC) contain one intron, and two genes (*clp*P, *ycf*3) have two introns; 18 genes (*rpl*2, *rpl2*3, *ycf*2, *ycf1*5, *ndh*B, *rps*7, *rps*12, *trn*I-CAU, *trn*L-CAA, *trn*V-GAC, 16S rRNA, *trn*I-GAU, *trn*A-UGC, *trn*R-ACG, *trn*N-GUU, 23S rRNA, 4.5S rRNA, 5S rRNA) have duplicates and five (*ycf*68, *orf*42, *orf*56, *ycf*1, *rps*19) partial genes (putative pseudogenes) in the IR regions. All assessed cpDNAs have collinear gene content and arrangement. The main difference among the three morphotypes is the inversion of *pet*N and *psb*M genes position in *U. amethystina* yellow in comparison to other *Utricularia* (Figure 2).

### 2.2. Repeats and Chloroplast Microsatellites (cpSSR)

**Figure 3:**
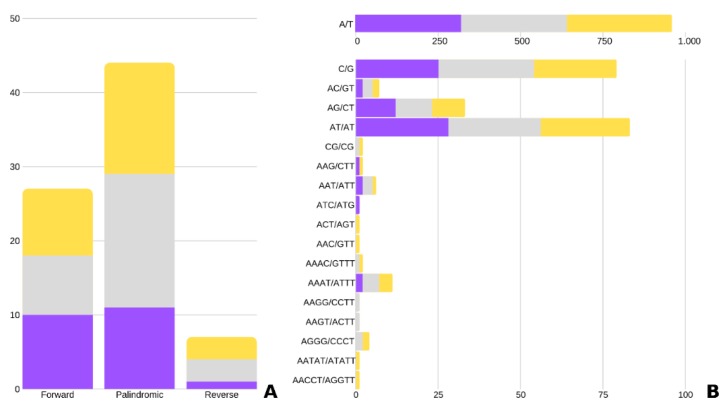
Quantity of repeats in *Utricularia amethystina*. (**A**) Long repeats. (**B**) Simple sequence repeats (cpSSRs). Purple, yellow, and gray bars denote *U. amethystina* purple, yellow, and white morphotypes, respectively. (Additional information can be found in Supplementary Table S5).

REPuter identified 22, 29, and 27 repeats in *U. amethystina* purple, white, and yellow, respectively (Figure 3A). Most were characterized as forwarding and palindromic repeats. The repeats were mainly distributed among the *ycf*2, *rpo*C1 and *trn*S-GCU, *ycf*3, *ndh*A genes, and *rbc*L*-acc*D, and *rps*12*-trn*V-GAC intergenic region (Supplementary Tables S2–S4). The identified microsatellite (cpSSR), vary from 7 to 369-bp for *U. amethystina* purple, 7 to 264-bp for *U. amethystina* yellow, and 7 to 245-bp for *U. amethystina* white (Figure 3B). The amount of identified cpSSR repeats are similar within all morphotypes, with the mono- (346, 350, 343), and dinucleotide repeats as the most abundant (42, 43, 40), followed by tri- (4, 3, 4) and tetra- repeats (2 for *U. amethystina* purple and 10 for white and 7 for yellow). Interestingly, penta- nucleotides were only found in *U. amethystina* yellow, and hexa- repeats were not found in any morphotypes (Supplementary Table S5).

### 2.3. Interspecific Comparison

**Figure 4:**
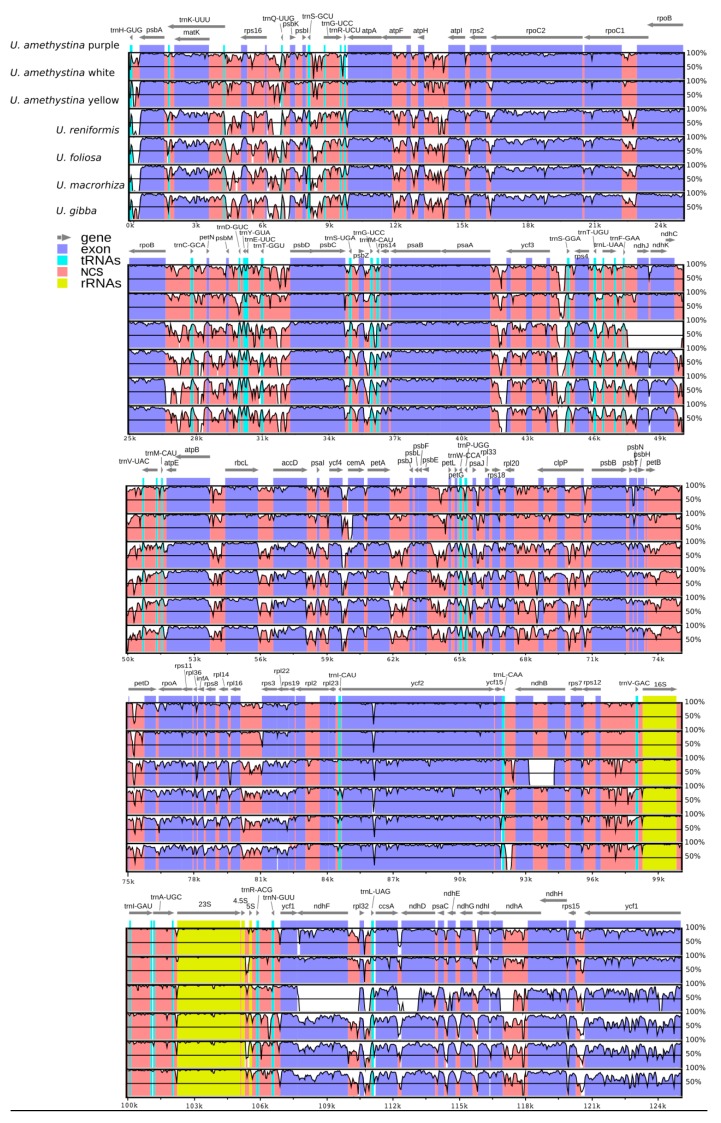
mVISTA identity plot based in Shuffle-LAGAN alignment for *Utricularia amethystina* morphotypes and previously reported chloroplasts of other *Utricularia* species. *U. amethystina* purple was used as a reference. NCS denotes non-coding sequence.

The interspecific chloroplast genome divergence among all available *Utricularia* cpDNAs, using *U. amethystina* purple as a reference, showed that *U. amethystina* specimens have a high degree of synteny; however, for *ycf*1 and non-coding sequences, they are highly divergent (<50% identity; Figure 4). Furthermore, the coding regions *ccs*A, *mat*K, *rpo*C2, *rpo*C1, *rps*19, *ycf*1, *ycf*2; the introns *atp*F and *rps*16; and most intergenic spacers, such as *trn*K-*rps*16, *rps*16-*trn*Q, *psb*K-*psb*I, *trn*L-*trn*F, and *trn*H-*psb*A, showed high levels of variation that can be used for phylogenetic and DNA-barcoding studies (Figure 4).

**Figure 5:**
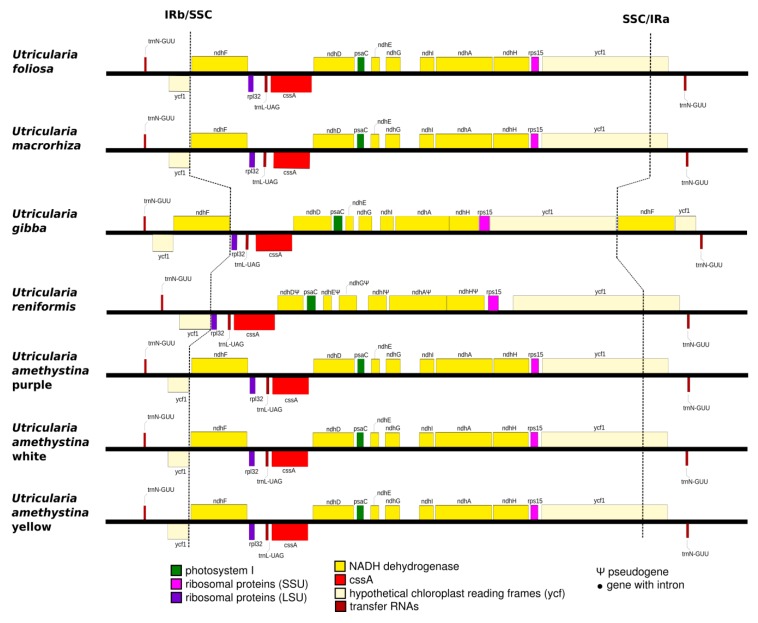
Boundaries between SSC regions of all previously published *Utricularia* species. cpDNA regions color denote different chloroplast gene families.

The SSC regions of *Utricularia amethystina* morphotypes are similar to most *Utricularia*, except for *U. reniformis*, due to the deletion of *ndh*s genes (Figure 5), and *U. gibba*, which have an extra copy of *ycf*1 and *ndh*F.

### 2.4. Intraspecific Comparison: Species Polymorphism

**Figure 6:**
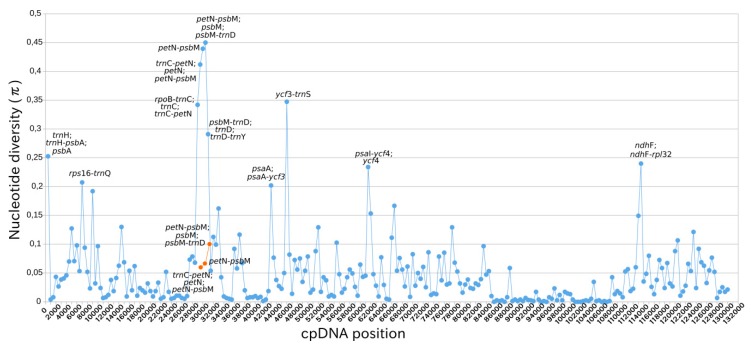
Nucleotide diversity (π) for the three *Utricularia amethystina* morphotypes. Each blue dot represents the nucleotide diversity per 500 bp. Orange dots denote the nucleotide diversity for the un-inverted region in *U. amethystina* yellow (*psb*M-*pet*N).

The nucleotide diversity (π) analyses indicated that the IRs exhibited lower variability than LSC and SSC regions (Figure 6). There are twelve spots of chloroplast genome regions that showed remarkably higher π values (>0.02), including nine gene (*trn*H, *psb*A, *trn*C, *pet*N, *psb*M, *trn*D, *psa*A, *ycf*4 and *ndh*F) and 12 intergenic regions (*trn*H-*psb*A, *rps*16-*trn*Q, *trn*C-*pet*N, *rpo*B-*trn*C, *pet*N-*psb*M, *trn*C-*pet*N, *psb*M-*trn*D, *trn*D-*trn*Y, *psa*A-*ycf*3, *ycf*3-*trn*S, *psa*I-*ycf*4, *ndh*F-*rpl*32). (More details in Supplementary Table S6.)

### 2.5. Chloroplast Expression

**Figure 7:**
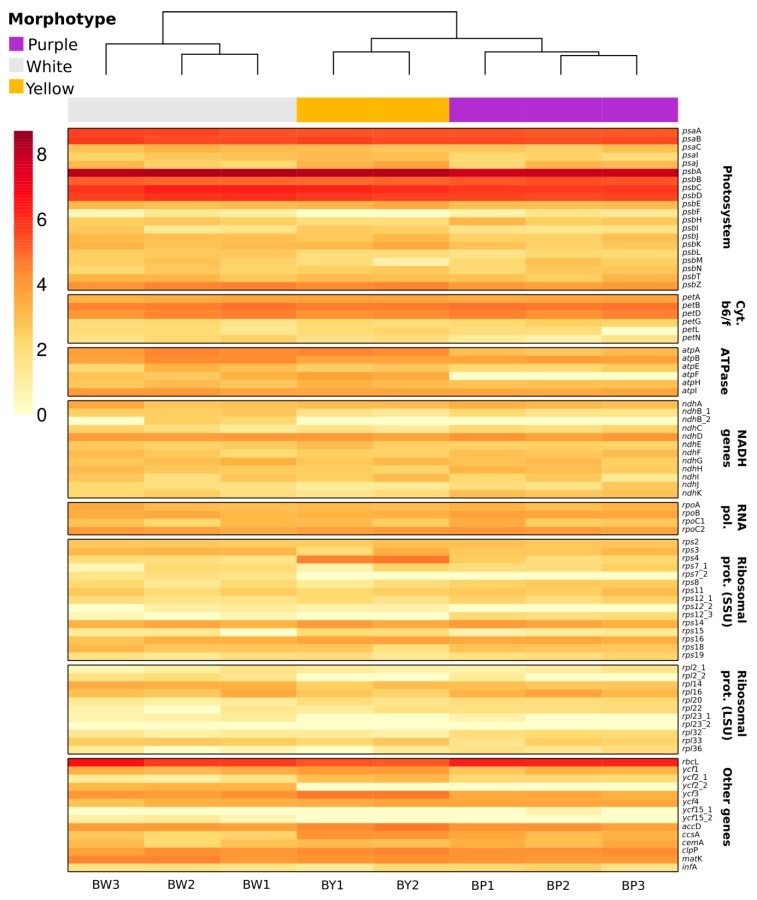
Heat map representation of *Utricularia amethystina* cpDNA transcript level. The underscore “_1” and “_2” denotes each gene duplicate. The *rps*12 is a duplicated trans-spliced gene, therefore it was analyzed in three parts.

RNAseq clustering analyses indicated distinct expression profiles for each *Utricularia amethystina* morphotype. The *psb*A and *rbc*L genes showed much higher levels of expression, followed by *psa*A, *psa*B, *psb*B, *psb*C, and *psb*D, in comparison to other genes in all samples (Figure 7; Supplementary Table S7).

### 2.6. RNA Edit

**Figure 8:**
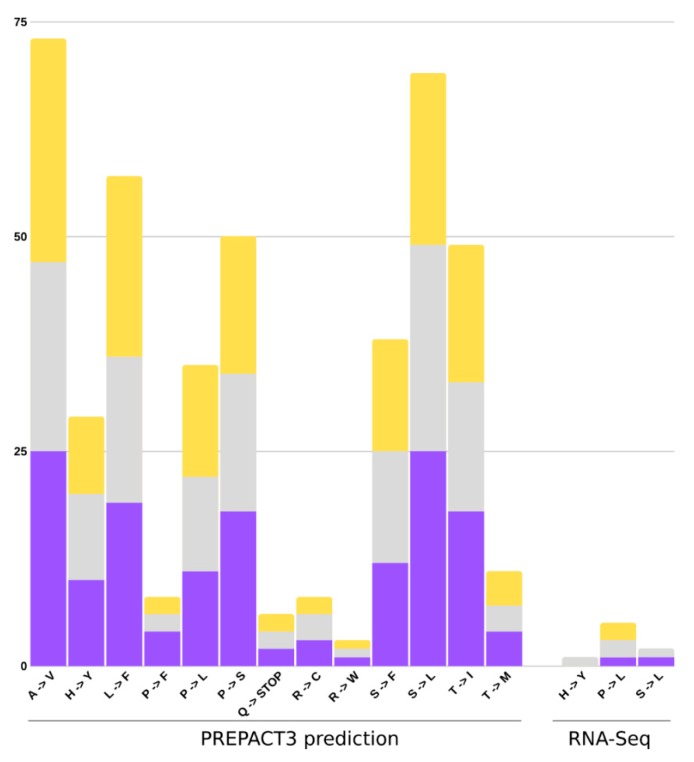
Quantities of amino acid changes from the prediction and RNA-Seq data of RNA-editing sites for each *Utricularia amethystina* morphotype. Purple, yellow, and gray bars denote *U. amethystina* purple, yellow, and white morphotypes, respectively.

The RNA editing analyses were carried out using the PREPACT3 tool, and in-house script to search for validation of editing sites using RNA-Seq mapped reads. The PREPACT3 predicted 154 sites for *U. amethystina* purple, 140 for *U. amethystina* white, and 146 for *U. amethystina* yellow. Most amino acid changes are shared between populations, and comparison between *Utricularia amethystina* morphotypes showed 22 genes with the same editing sites and 15 genes with differences in the quantity and amino acid composition changes (for more information see Supplementary Tables S8–S10). According to the results, there are 13 types of amino acid transitions in the three *U. amethystina*. The most prevalent nonsynonymous substitutional changes occurred between Alanine to Valine and Serine to Leucine, followed by Leucine to Phenylalanine, Proline to Serine, Threonine to Isoleucine, Serine to Phenylalanine, Proline to Leucine, Histidine to Tyrosine, Threonine to Methionine, Proline to Phenylalanine, Arginine to Cysteine, Glutamine to Stop codon, and Arginine to Tryptophan (Figure 8). For RNA-Seq-based results, only three nonsynonymous amino acid substitutions were found (Figure 8; Table 2).

The results from RNA-Seq data showed eight nonsynonymous and eight synonymous sites for the three *U. amethystina* morphotypes. Regarding nonsynonymous sites, *U. amethystina* purple and yellow have *rps*14 and *pet*B genes edition, and for *U. amethystina* white, edition sites were two in *rps*14, *pet*B, and *ndh*B (Table 2). The same edited sites in the same gene position were found in *pet*B for all *U. amethystina* samples. In addition, the *rps*14 gene from purple is the same as *rps*14 in position 36,572 for white, and *rps*14 gene from yellow is the same as the *rps*14 in position 36,497 for white.

### 2.7. Phylogeny

**Figure 9:**
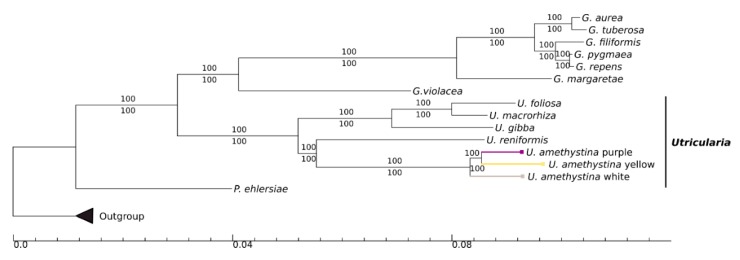
Bayesian inference tree for available Lentibulariaceae chloroplast genomes and studied *Utricularia amethystina*. The values above branches denote the posterior probabilities and below maximum likelihood bootstrap. The branch colors correspond to each *U. amethystina* morphotype flower color. (*G*. = *Genlisea*; *P*. = *Pinguicula*; *U*. = *Utricularia*). The scale represents the expected changes per site.

The phylogenetic tree based on available chloroplast genomes of 15 Lentibulariaceae specimens is shown in Figure 9. Both maximum likelihood (ML) and Bayesian inference (BI) trees exhibited identical phylogenetic topologies, and support values (bootstrap for ML and posterior probabilities for BI) are 100% for all clades. The Lentibulariaceae is known to be monophyletic, and *Pinguicula* is a sister clade to *Utricularia*-*Genlisea*. The results show that the *Utricularia* genus is monophyletic and the three *U. amethystina* are closely related to *U. reniformis*. Also, the *U. amethystina* yellow is sister to purple, with white as having the same common ancestor (Figure 9).

## 3. Discussion

Due to the plasticity in corolla shape and color, *Utricularia amethystina* is one of the most polymorphic species within the *Utricularia* genus. This polymorphism resulted in a historically taxonomic complicated group with its systematics only partially resolved to date. During the last decades, several efforts attempted to separate the different *U. amethystina* morphotypes into different species, yet without much success [7,12,13].

In this study, we analyzed the cpDNAs of three morphologically distinct *Utricularia amethystina* from different populations: the purple, white, and yellow morphotypes, aiming to detect intra- and interspecific variations and phylogenetic signals and provide new cpDNA regions for evolutionary studies. In addition, we evaluated the transcription and RNA editing sites for *U. amethystina* populations.

In an attempt to diminish the environmental conditions bias, we have collected the specimens from close populations (~2.8 km between purple and white, 0.2 km between white and yellow, and 2.82 km between purple and yellow. The specimens of *U. amethystina* cpDNA have a typical quadripartite structure present in most land plants and have a similar organization and GC content to other *Utricularia* [24,27].

Among the three *Utricularia amethystina* morphotypes, we found an inversion between the *pet*N and *psb*M genes in *U. amethystina* yellow, representing the first known gene inversion in LSC region identified in Lentibulariaceae chloroplast genomes. Indeed, the same inversion was detected in the chloroplast genome of species of Cannabaceae [30], and microstructural short inversions of 10 bp were also found in the *pet*N-*psb*M region in *Solanus* species [31]. Some comparative cpDNA studies have also identified structural mutations in monilophyte chloroplast genomes, including as many as six inversions and some gene losses (e.g., in [32,33,34]).

In general, chloroplast deletions/losses are observed among Lentibulariaceae. Indeed, *Utricularia reniformis* suffered a major SSC region retraction due to the losses of NAD(P)H-dehydrogenase (*ndh*) complex genes [27]. In contrast, all other sequenced *Utricularia* cpDNAs have complete *ndh*s gene complexes. These chlororespiratory genes are *ndh*A, B, C, D, E, F, G, H, I, J, and K, and encode subunits of the NADH dehydrogenase complex in plant chloroplast genomes that play a role in plant signaling in the photosynthesis reaction [35] and the reduction and oxidation of plastoquinones [36]. As *U. reniformis* is a terrestrial species, it has been proposed that possibly all terrestrial species of *Utricularia* may lack members from the *ndh* genes complex [27,28]. However, *U. amethystina* is terrestrial, and all three morphotypes retain all plastid *ndhs* complex genes. Therefore, our results now suggest that the *ndh*s in terrestrial *Utricularia* were independently lost and regained, thus refuting the hypothesis (at least for *Utricularia*) that terrestrial species have experienced the loss of *ndh*s genes.

Chloroplast repeats are important regions for replication and DNA stability [37]. Microsatellites or SSRs are tandem repeats of 1–6 base pairs units long that can be used as genetic markers [38]. They are most commonly found in plants and due to genetic variation in the number of tandem repeats units. Therefore, as they produce polymorphism detectable with PCR-based methods banding pattern and genotyping, the SSRs are widely used in population genetics and evolutionary studies [39]. *Utricularia amethystina* has high amounts of mononucleotide repeats in the cpSSR, which is similar to other angiosperms, such as *Arabidopsis thaliana* [40], and other Lentibulariaceae [24,25,27]. Previous results for *Utricularia* indicated that most of SSR were found in coding regions for *U. gibba*, *U. macrorhiza*, *Genlisea margaretae*, and *Pinguicula ehlersiae*. However, for *U. reniformis*, more cpSSR were found in non-coding regions. In *U. amethystina*, long repeats have similar quantities between populations, and as seen in other *Utricularia*, most of them are in coding regions [24,27], an uncommon fact for other angiosperms chloroplast genomes (e.g., see [41]), which could indicate high rates of recombination and rearrangement, as discussed in Silva et al. (2016) [27]. Although long repeats could be the cause for gene rearrangements, we could not find repeats in flanking regions of the genes *psb*M and *pet*N in *U. amethystina* yellow. Therefore, this indicates that other evolutionary forces were involved with the observed inversion of these genes in this species.

For some *Utricularia*, DNA barcoding approaches have been considered a difficult task to perform. For instance, the DNA-barcoding markers, such as ITS, *rbc*L, and *mat*K, could not discriminate all *Utricularia* accessions at the species and population level due to their low level of polymorphism (e.g., *Utricularia* sect. *Utricularia* in Astuti et al., 2019 [42]). Furthermore, *rps*16, *trn*L-F, and *trn*D-T markers cannot discriminate *U. amethystina* populations [13]. Therefore, it is important to explore regions with high variability at inter- and intraspecies levels that represent potentially useful markers for future studies. Using mVISTA results for the interspecific divergence analysis, it is noticeable that the LSC and SSC regions are more variable than IR regions, corroborating with the results found for identity analyses with *Genlisea* species [25] and other angiosperms [41]. The results showed highly variable regions between the different species, mostly represented by intergenic spacers, such as *trn*H-*psb*A, *trn*K-*rps*16, and *rps*16-*trn*Q, which could be used for interspecies identification.

It is previously proposed that populations from closely related environments should be less divergent if they are of the same species. However, we observed high intraspecific chloroplast sequence variability, although geographical sampling covered a restricted area. Among the regions with high nucleotide diversity and intraspecific variations, there is the intergenic spacer, *trn*H-*psb*A, which is already being used as DNA barcoding in many studies [43]. This study also revealed spots that can be used for populations and phylogenetic analyses due to high variability, such as the genes *trn*H, *psb*A, and intergenic regions, such as *trn*H-*psb*A, *ycf*3-*trn*S, and *rps*16-*trn*Q (see more in the Results section and Supplementary Material S6). Nevertheless, the spots of diversity near the genes *pet*N and *psb*M should be avoided due to low primer annealing considering that the region could be inverted, as seen in *U. amethystina* yellow.

The preparation of paired-end libraries was enriched for polyadenylated transcripts which causes the instability of organelle transcripts, therefore there is probably underrepresentation of transcripts [44]. However, we were able to observe that almost all chloroplast protein-coding genes are expressed in all sampled flower tissue of *U. amethystina*, except for *ycf*15, both *rpl*23 duplicated genes in *U. amethystina* purple and yellow, and the *atp*F gene in *U. amethystina* purple (Supplementary Table S7).

The expression profile is similar between samples of the same morphotypes and even expression profile clustering corresponds to the phylogenetic hypothesis proposed in this research. The *rbc*L gene was one of the most highly expressed genes and encodes for one of the most abundant enzymes in nature, the large subunit of ribulose-1-5-biphosphate carboxylase [45]. This protein is involved in fixing CO_2_ and photorespiration [46]_._ Moreover, high levels of gene expression were found in Photosystem I (PSI) and II genes (PSII), such as *psa*A and *psa*B, and *psb*A, *psb*B, *psb*C, and *psb*D, these proteins are involved in photosynthesis [47]. Studies of barley leaf activities showed that dark-grown plants were deficient in PSI and PSII proteins [48]. Moreover, Klein et al. (1988) showed that the elongation of translation in *psa*A, *psa*B, *psb*A, and *rbc*L are regulated by light [49,50]. Therefore, considering that the corollas were collected during the day, our results are congruent with the hypothesis of a protein exhibiting light-induced translation.

Interestingly, the *pet*N and *psb*M genes are expressed in all *Utricularia amethystina* biological samples, indicating that, the inversion observed in *U. amethystina* yellow did not affect the expression of these genes.

RNA editing sites are common features of a plant chloroplast. These mutations usually occur from C-to-U in mRNA molecules, and thus have an important role in the differential amino acid generation that can lead to different proteins originated from the same gene [51]. RNA-Seq-based results showed that there is a sum of eight editing sites for all *U. amethystina* morphotypes (Table 2).

The PREPACT3′s prediction showed that most nonsynonymous substitutions were characterized as Alanine to Valine and Serine to Leucine. Both lead to protein variations (Table 2, Tables S8–S10), whereas amino acid changes from Alanine to Valine, Histidine to Tyrosine, Leucine to Phenylalanine, Proline to Phenylalanine, Proline to Leucine, Proline to Serine, Arginine to Tryptophan, Threonine to Isoleucine, and Threonine to Methionine result in no physicochemical properties changes in protein. In addition, the Arginine to Cysteine, Serine to Leucine, and Serine to Phenylalanine mutations can modify protein formation due to hydrophilic (Serine and Arginine) to hydrophobic (Leucine, Phenylalanine, and Cysteine) molecule changes [52,53]. Moreover, PREPACT3 has predicted that the genes *rps*2 and *rpl*32 can be edited from Glutamine into a Stop codon, and despite they could be polycistronic genes as in other plants [54], these genes are still transcribed according to RNA-Seq data.

The presented evolutionary history, based on whole chloroplast DNA genomes, and reconstructed by ML and BI approaches supported the same relationship within the Lentibulariaceae when compared with one or few loci–loci analyses (Figure 8) [2,55]. These analyses and many other studies indicated that *Utricularia amethystina* can be paraphyletic [13]. However, in this study, despite the differences in the specimen, they are still a monophyletic taxon. This indicated that *U. amethystina* morphotypes have a common ancestry, but the sampling of other species from sect. *Foliosa* (*U. tricolor* and *U. tridentata*) and species from the close phylogenetically related sect. *Psyllosperma* would be necessary to shine this issue.

Our results support that the sampling based on three different morphotypes proved to be insufficient to allow firm conclusions on the *U. amethystina* species separation, considering we sampled one individual per morphotype. However, the scenario presented here based on chloroplast genomes suggests that *U. amethystina* morphotypes may be different species as previous studies based on morphometric approach [12] and phylogeny with few loci [13], but with more populations, have suggested.

Moreover, the comparative and functional analyses provided by this study bring new insights into the *Utricularia* chloroplast genome architecture, in particular, the evolutionary history of *ndh* complex genes and other important photosynthesis-related genes. Taken together, these results prove that we are just in the beginning for the understanding of the evolution of chloroplast photosynthesis machinery in the Lentibulariaceae.

## 4. Materials and Methods

### 4.1. Sampling and DNA Extraction

The three *Utricularia amethystina* morphotypes were collected from natural populations geographically close to each other (~2.8 km between purple and white; 0.2 km between white and yellow; and 2.82 km between purple and yellow). The samples were preserved in silica gel and stored at room temperature. Vouchers were deposited at Universidade Estadual Paulista (UNESP) in the Herbarium JABU (Table S1). The total genomic DNA was extracted from approximately 0.1 µg of flowers with Qiagen DNeasy Plant Mini extraction kit (Qiagen, Hilden, Germany) following manufacturer’s protocol. The quality and quantity of DNA were estimated with Nanodrop 2000 (Thermo Scientific, MA, USA) and Qubit fluorometer (Life Technologies, CA, USA), respectively.

### 4.2. Organellar Genome Sequencing, Assembly and Annotation

Sequence libraries were quantified using Bioanalyzer 2100 (Agilent, CA, USA). The paired-end libraries were prepared using Illumina library preparation manufacturer’s protocol, and genomic DNA of 2 × 100 bp and insert size of ~200 bp was sequenced using Illumina MiSeq platform (Illumina, San Diego, CA, USA)

The produced paired-end reads filtered for adapters, low-quality bases (Phred score Q > 24) and size (length cutoff for 50 bp), and possible contaminants using Trimmomatic v. 0.38 [56]. The resulting paired-end reads were mapped in the search for discarding mitochondria and nuclear reads with Bowtie2 v.2.2.3 [57] using very sensitive local and -N 1 parameters and *Utricularia* spp. (NC_021449, KY025562, and KT336489) chloroplasts as reference genomes. The resulting reads were assembled using SPAdes v. 3.7.1 [58] and regions with assembly uncertainties were extended using iterative read mapping performed using MITObim v.1.8 [59].

The organelles genomes were primarily annotated using DOGMA [60], cross checked with GeSeq [61], and start and stop codons were adjusted manually for annotation refinements. The tRNAs were annotated using tRNA-scan [62], implemented in DOGMA and Aragorn [63]. The rRNAs were annotated using RNAmmer and BLASTn searches with available *Utricularia* cpDNA genomes. The cp genome map was constructed using the Organellar Genome Draw program [64].

### 4.3. Repeats and SSR Analyses

To avoid redundant results, only one IR of each *Utricularia amethystina* cpDNA was used and direct, forward, reverse, and palindromic repeats were identified using REPuter [65] with a minimal size of 30 pb and Hamming distance of 3. Simple sequence repeats (SSR) were detected using MISA-web [66] by setting the minimum number of repeats to 7, 4, 4, 3, 3, and 3, for mono-, di-, tri-, tetra-, penta-, and hexanucleotides, respectively.

### 4.4. Phylogenetic Reconstruction

Sequence alignment for all published and available Lentibulariaceae cpDNA genomes (Species and accession number, respectively: *Utricularia foliosa*, KY025562; *U. gibba*, KC997777; *U. reniformis*, KT336489; *U. macrorhiza*, HG803177; *Pinguicula ehlersiae,* HG803178, *Genlisea aurea*, MF593121; *G. violacea*, MF593126; *G. tuberosa*, NC_037082; *G. filiformis*, MF593122; *G. pygmaea*, MF593123; *G. repens*, MF593124; *G. margaretae*, HG530134) were performed with online MAFFT v.7 [67] with default parameters. The phylogenetic tree reconstruction was performed using the Bayesian inference (BI) and maximum likelihood (ML) approaches under the best-of-fit model GTR+G+I, in accordance with Akaike Information Criterion (AIC), assessed by Mr.ModelTest v.2.4 [68]. For BI, set for the substitution model accordingly, we employed Mr.Bayes v.3.2.6 software with 5 × 10^7^ generations sampled for each 1000 generations using two runs and four chains, until the average standard deviation of split frequencies became less than 0.01, beginning with random trees. The initial trees were discarded after reaching stationary (~25%). For ML we used the software RAxML v.8.2.10 [69] with 1000 bootstrap pseudoreplicates. The analyses were performed using implementations within CIPRES Science Gateway v.3.3 (https://www.phylo.org/) and cladograms were edited using TreeGraph v.2.15 beta [70]. The species *Tectona grandis* (NC020098), *Sesamum indicum* (NC016433), and *Tanaecium tetragonolobum* (NC027955) were used as outgroup.

### 4.5. Intraspecific Polymorphism Analyses

For polymorphism analyses, the *Utricularia amethystina* chloroplasts were aligned using MAFFT v.7, with default parameters. Based on the cpDNA multiple alignments, polymorphism analysis was conducted for coding genes, introns, and intergenic spacers. The nucleotide diversity was calculated using Tassel v.5.2.54 [71] with a sliding window of 500 bp length.

### 4.6. Interspecific Comparison

Using the *Utricularia amethystina* purple as a reference and previously published *Utricularia (Utricularia foliosa*, KY025562; *U. gibba*, KC997777; *U. reniformis*, KT336489; *U. macrorhiza*, HG80317), the identity of cpDNA was assessed using mVISTA online software (http://genome.lbl.gov/vista/mvista/submit.shtml) with Shuffle-LAGAN Mode.

### 4.7. RNA Extraction, Sequencing and RNA Editing Site Analyses

The corollas of *Utricularia amethystina* were stored in RNAlater^®^ (Thermo Fisher Scientific, MA, USA) from each analyzed population and were used as plant tissues for RNA-Seq. The corollas (~5 per specimen) were pooled in three replicates for *U. amethystina* white and purple and two for *U. amethystina* yellow, and total RNA was extracted using PureLink RNA MiniKit (Thermo Fisher Scientific, MA, USA), according to manufacturer’s protocol. The extracted RNA was analyzed with Agilent 2100 Bioanalyzer and Qubit 2.0 Fluorometer for quality and quantity assessment, and only samples with RNA Integrity Number (RIN) > 7.0 were used for the sequencing.

The eight libraries (3 libraries for each *U. amethystina* purple and white and 2 for *U. amethystina* yellow) were constructed following the TruSeq Stranded mRNA LS Protocol sample preparation protocol. The paired-end (2 × 100 pb) sequencing was performed in one lane on an Illumina platform (HiSeq 2500) following supplier-provided protocols (Illumina, San Diego, CA, USA).

The raw sequencing data, was preprocessing with high stringency using the following steps. (1) For the 3′ end, the adapter and low-quality reads were removed using Scythe (https://github.com/vsbuffalo/scythe; default parameters, except for -n 5 and -M 15); (2) for the 5′ end, the removal of adapter and low quality reads were performed with Cutadapt [72]; default parameters, except for –overlap 5; –minimum-length = 15; --times = 2); (3) to filter reads with more than 30% of unknown base (Ns), polyA/T tails we used the software Prinseq [73].

Filtered RNA-seq reads were mapped against the assembled chloroplast genome using STAR version 2.7.2a [74], using default parameters except for adjusted parameters to perform an end-to-end mapping, diminish multiple mapping of the same reads, minimum and maximum size of introns and the number of allowed mismatches (--outFilterMultiMapMax = 3; --outFilterMismatchNmax = 2; --outFilterMismatchNoverLmax = 0.1; --outSJfilterReads = Unique; --alignEndsType = EndtoEnd; --alignIntronMin = 70; --alignIntronMax = 2500). To estimate differential transcripts abundance between biological replicates, normalized count data was obtained using relative log expression (RLE) method in DESeq2 version 3.9 [75] and results were showed following with log2(norm. counts+1). The *rps*12 is a duplicated trans-spliced gene, therefore it was analyzed in three parts and “_2” and “_3” represent the duplicated regions. The putative RNA edit sites were predicted following PREPACT3 software [76] with BlastX searches (using default parameters) against the *Nicotiana tabacum*, as reference. The prediction results were compared with the results obtained with an in-house script that counts the number of editing sites according to the previous STAR mapped reads, except for the number of mapped reads, which was set to 1 (only uniquely mapped reads). All of the sites were inspected for C to U nucleotide substitutions by a custom Perl script, with the use of the following parameters; presence in at least two of the biological replicates, editing set with a minimum coverage of 10×.

## Figures and Tables

**Table 1:** The summary of characteristics in *Utricularia amethystina* chloroplast genomes. Between parentheses the percentage that represents each part in comparison to the cpDNA genome.

U. amethystina Morphotypes	Purple	White	Yellow
Genbank accession number	MN223721	MN223722	MN223720
Genome size (bp)	150,115	150,332	150,020
Large single copy (LSC) length (bp)	82,388 (54.9%)	82,561 (54.9%)	82,256 (54.8%)
Small single copy (SSC) length (bp)	16,969 (11.3%)	17,070 (11.4%)	16,870 (11.3%)
Inverted repeats (IR_a_+IR_b_) length (bp)	25,399 (34.7%)	25,350 (33.8%)	25,447 (34.0%)
Noncoding regions (bp)	40,199	41,901	41,259
Intronic regions(bp)	18,052	19,260	20,280
%GC	37.5	37.5	37.7
Coverage	84×	119×	123×

**Table 2:** RNA editing sites predicted using RNAseq data in *Utricularia amethystina* cpDNA. The editing level is given in percentage and is showed for each biological replicate. * denotes amino acids with a change in physicochemical composition.

Morphotype	Gene	Genome (cpDNA)		Codon		Codon Position	Amino Acid		Editing Level		
									(Each Biorep)		
**Purple**		**Position**	**Strand**	**from**	**to**		**from**	**to**	**P1**	**P2**	**P3**
	*rps*14 *	36,640	-	UCA	UUA	2	S	L	96	100	100
	*pet*B	74,797	+	CCA	CUA	2	P	L	100	100	97
**White**									**W1**	**W2**	**W3**
	*ndh*B	138,239	+	CCA	CUA	2	H	Y	92	82	0
	*ndh*D	113,305	-	CUA	UUA	1	Synonym		100	0	100
	*rbc*L	55,651	+	GCC	GCU	3	Synonym		100	100	0
		55,777	+	AUC	AUU	3	Synonym		100	100	0
	*rps*14 *	36,572	-	UCA	UUA	2	S	L	38	14	75
		36,497	-	CCA	CUA	2	P	L	100	0	72
		36,553	-	AAC	AAU	3	Synonym		100	100	0
	*psb*B	72,140	+	GGC	GGU	3	Synonym		100	100	0
		71,372	+	UAC	UAU	3	Synonym		100	0	100
	*psa*A	39,370	-	AUC	AUU	3	Synonym		100	100	100
	*pet*B	74,816	+	CCA	CUA	2	P	L	100	0	97
**Yellow**									**Y1**	**Y2**	-
	*rps*14	36,439	-	CCA	CUA	2	P	L	69	71	-
	*ccs*A	111,950	+	CUA	UUA	1	Synonym		18	16	-
	*pet*B	74,948	+	CCA	CUA	2	P	L	100	100	-

## Supplementary Materials

Supplementary materials can be found at https://www.mdpi.com/1422-0067/20/24/6130/s1.
