# Supplementary material for: Intraspecific Variation within the Utricularia amethystina Species Morphotypes Based on Chloroplast Genomes
Source: Int J Mol Sci. 2019 Dec 5;20(24):6130. doi: 10.3390/ijms20246130 (PMC6940893; doi:10.3390/ijms20246130)
Supplement: Supplementary file 1 [file ijms-20-06130-s001.zip › Supplementary_Table_S1.docx]

**Supplementary Table S1.** Samples and chloroplast descriptions.

| **Species morphotypes** | **purple** | **white** | **yellow** |
| --- | --- | --- | --- |
| Voucher number | VM1998 | CM131 | VM2032 |
| Locality | Alto Paraíso de Goiás - GO - Brazil | Alto Paraíso de Goiás - GO - Brazil | Alto Paraíso de Goiás - GO - Brazil |
| Coordinates | S 13.96255º  W 47.47467º | S 13.94738º  W 47.49458º | S 13.94536º  W 47.49447º |
| Habitat | Sandy soil | Sandy soil with running water | Sandy soil with running water |
| Number of genes | 137 | 137 | 137 |
| Number of unique protein-coding genes | 39 | 39 | 39 |
| Number of tRNA genes | 30 | 30 | 30 |
| Number of rRNA genes | 4 | 4 | 4 |
| Number of genes with intron | 18 | 18 | 18 |
