# Supplementary material for: Intraspecific Variation within the Utricularia amethystina Species Morphotypes Based on Chloroplast Genomes
Source: Int J Mol Sci. 2019 Dec 5;20(24):6130. doi: 10.3390/ijms20246130 (PMC6940893; doi:10.3390/ijms20246130)
Supplement: Supplementary file 1 [file ijms-20-06130-s001.zip › Supplementary_Table_S2-S4.docx]

**Table S2.** Sequence repeats in *Utricularia amethystina* purple. Type, length, region, location and plastome quadripartite region for each repeat are indicated. (Type: F = Forward repeat; P= Palindromic repeat; T = Tandem repeat.)

| **Repeat 1** | **Type** | **Length (bp)** | **Repeat 2** | **Gene** | **Location** | **Region** |
| --- | --- | --- | --- | --- | --- | --- |
| 86083 | F | 42 | 86122 | *ycf2* | CDS | IR |
| 72841 | P | 45 | 72841 | *psbT-psbN* | IGS | LSC |
| 46321 | P | 47 | 76321 | *petD-rpoA* | IGS | LSC |
| 32045 | P | 39 | 32045 | *trnT(GGU)-psbD* | IGS | LSC |
| 59708 | P | 43 | 59708 | *ycf4-cemA* | IGS | LSC |
| 44438 | P | 35 | 44540 | *ycf3-trnS(GGA)* | IGS | LSC |
| 8031 | P | 30 | 44863 | *trnS(GCU)-trnS(GGA)* | IGS | LSC |
| 43233 | F | 36 | 96430 | *ycf3; rps12-trnV(GAC)* | CDS;IGS | IR |
| 43236 | F | 36 | 117003 | *ycf3; ndhA(intron)* | CDS;intron | LSC;SSC |
| 55907 | P | 36 | 55907 | *rbcL-accD* | IGS | LSC |
| 96428 | F | 38 | 116998 | *rps12-trnV(GAC);ndhA(intron)* | IGS;intron | IR;SSC |
| 38203 | F | 35 | 40427 | *psaB;psaA* | CDS | LSC |
| 97051 | F | 32 | 97079 | *rps12-trnV(GAC);rps12-trnV(GAC)* | IGS | IR |
| 86101 | P | 31 | 86135 | *ycf2* | CDS | IR |
| 112249 | P | 30 | 112279 | *ccsA-ndhD* | IGS | SSC |
| 8029 | F | 32 | 34946 | *trnS(GCU)* | tRNA | LSC |
| 35857 | P | 31 | 35857 | *psbZ-trnG(GCC)* | IGS | LSC |
| 89716 | F | 31 | 89734 | *ycf2* | CDS | IR |
| 22427 | R | 30 | 22440 | *rpoC1* | CDS | LSC |
| 34948 | P | 30 | 44863 | *trnS(UGA)* | tRNA | LSC |
| 58465 | F | 30 | 110738 | *accD-psaI* | IGS | LSC |
| 87328 | F | 30 | 87370 | *ycf2* | CDS | IR |

**Table S3.** Sequence repeats in *Utricularia amethystina* yellow. Type, length, region, location and plastome quadripartite region for each repeat are indicated. (Type: F = Forward repeat; P= Palindromic repeat; T = Tandem repeat.)

| **Repeat 1** | **Type** | **Length (bp)** | **Repeat 2** | **Gene** | **Location** | **Region** |
| --- | --- | --- | --- | --- | --- | --- |
| 106893 | P | 491 | 124819 | *trnN-ycf1;ycf1* | IGS;CDS | IR;SC |
| 107201 | P | 427 | 124575 | *ycf1* | CDS | SSC |
| 106786 | P | 414 | 125003 | *trnN-ycf1;ycf1* | IGS;CDS | IR;SSC |
| 107385 | P | 243 | 124575 | *ycf1* | CDS | SSC |
| 106738 | P | 60 | 125233 | *trnN-ycf1;ycf1* | IGS;CDS | IR;SSC |
| 112254 | P | 44 | 112254 | *ccsA* | CDS | SSC |
| 72785 | P | 34 | 72785 | *psbB-psbT* | IGS | LSC |
| 50152 | F | 41 | 50175 | *ndhC-trnV(UAC)* | IGS | LSC |
| 38083 | F | 30 | 40307 | *ndhC-trnV(UAC);psaA* | IGS;CDS | LSC |
| 8033 | P | 36 | 44598 | *trnS(GCU)-trnG(UCC); trnS(GGA)* | IGS;tRNA | LSC |
| 43147 | F | 36 | 96289 | *ycf3(intron);rps12(intron)* | Intron | LSC;IR |
| 43150 | F | 36 | 116962 | *ycf3;ndhA(intron)* | Intron;CDS | LSC;SSC |
| 55808 | P | 36 | 55808 | *rbcL-accD* | IGS | LSC |
| 113904 | P | 36 | 113904 | *ndhD* | CDS | SSC |
| 96287 | F | 38 | 116957 | *rps12(intron);ndhA* | intron;CDS | IR;SSC |
| 107602 | P | 31 | 124570 | *ycf1* | CDS | SSC |
| 6748 | P | 32 | 6748 | *trnQ(UUG)-psbK* | IGS | LSC |
| 96913 | F | 32 | 96941 | *rps12-trnV(GAC)* | IGS | IR |
| 7766 | P | 34 | 73822 | *psbI-trnS(GCU);petB(intron)* | IGS;intron | LSC |
| 6741 | R | 31 | 6741 | *trnQ(UUG)* | tRNA | LSC |
| 41682 | R | 30 | 41693 | *psaA-ycf3* | IGS | LSC |
| 8031 | F | 32 | 34837 | *trnS(GCU)-trnG(UCC); trnS(GGA)* | IGS;tRNA | LSC |
| 89585 | F | 31 | 89603 | *ycf2* | CDS | IR |
| 22302 | R | 30 | 22315 | *rpoC1* | CDS | LSC |
| 30754 | P | 30 | 30772 | *trnT(GGU)* | tRNA | LSC |
| 34839 | P | 30 | 44598 | *trnS(UGA);trnS(GGA)* | tRNA | LSC |
| 87197 | F | 30 | 87239 | *ycf2* | CDS | IR |

**Table S4.** Sequence repeats in *Utricularia amethystina* white. Type, length, region, location and plastome quadripartite region for each repeat are indicated. (Type: F = Forward repeat; P= Palindromic repeat; T = Tandem repeat.)

| **Repeat 1** | **Type** | **Length (bp)** | **Repeat 2** | **Gene** | **Location** | **Region** |
| --- | --- | --- | --- | --- | --- | --- |
| 106916 | P | 685 | 124602 | *ycf1* | CDS | IR |
| 106971 | P | 630 | 124602 | *ycf1* | CDS | IR |
| 106893 | P | 491 | 124819 | *ycf1* | CDS | IR |
| 107201 | P | 427 | 124575 | *ycf1* | CDS | IR |
| 106786 | P | 414 | 125003 | *trnN(GUU)-ycf1;ycf1* | IGS;CDS | IR |
| 107385 | P | 243 | 124575 | *ycf1* | CDS | IR |
| 106738 | P | 232 | 125233 | *trnN(GUU)-ycf1;ycf1* | tRNA;CDS | IR |
| 112254 | P | 60 | 112254 | *ccsA; ccsA-ndhD* | CDS;IGS | SSC |
| 72785 | P | 44 | 72785 | *psbT* | CDS | LSC |
| 50152 | F | 34 | 50175 | *ndhC-trnV(UAC)* | IGS | LSC |
| 38083 | F | 41 | 40307 | *psaB;psaA* | CDS | LSC |
| 8033 | P | 30 | 44598 | *psbI-trnS(GCU);trnS(GGA)* | IGS;tRNA | LSC |
| 43147 | P | 36 | 96289 | *ycf3;rps12* | CDS | LSC |
| 43150 | F | 36 | 116962 | *ycf3;ndhA* | CDS | LSC;SSC |
| 55808 | P | 36 | 55808 | *rbcL* | CDS | LSC |
| 113904 | P | 36 | 113904 | *ndhD* | CDS | SSC |
| 96287 | F | 38 | 116957 | *rps12;ndhA* | CDS | LSC;SSC |
| 107602 | P | 31 | 124570 | *ycf1* | CDS | IR |
| 6748 | P | 32 | 6748 | *rps16;trnQ(UUG)* | CDS;tRNA | LSC |
| 96913 | F | 32 | 96289 | *rps12-trnV(GAC)* | IGS | IR |
| 7766 | P | 34 | 73822 | *psbK-psbI;petB(intron)* | IGS;intron | LSC |
| 6741 | R | 31 | 6741 | *rps16;trnQ(UUG)* | CDS;tRNA | LSC |
| 41682 | R | 30 | 41693 | *psaA-ycf3* | IGS | LSC |
| 8031 | F | 32 | 34837 | *psbI-trnS(GCU);psbC-trnS(UGA)* | IGS | LSC |
| 89585 | F | 31 | 89603 | *ycf2* | CDS | IR |
| 22302 | R | 30 | 22315 | *rpoC1* | CDS | LSC |
| 30754 | P | 30 | 30772 | *trnE(UUC)-trnT(GGA)* | IGS | LSC |
| 34839 | P | 30 | 44598 | *psbC-trnS(UGA);trnS(GGA)* | IGS;tRNA | LSC |
| 87197 | F | 30 | 87239 | *ycf2* | CDS | IR |
