# Supplementary material for: Intraspecific Variation within the Utricularia amethystina Species Morphotypes Based on Chloroplast Genomes
Source: Int J Mol Sci. 2019 Dec 5;20(24):6130. doi: 10.3390/ijms20246130 (PMC6940893; doi:10.3390/ijms20246130)
Supplement: Supplementary file 1 [file ijms-20-06130-s001.zip › Supplementary_Table_S5.docx]

**Table S5.** Amount of sequence repeats in *Utricularia amethystina* purple. white, and yellow morphotypes.

| **SSR/Species morphotypes** | **purple** | | **white** | | **yellow** | |  |
| --- | --- | --- | --- | --- | --- | --- | --- |
| A/T | 321 | | 321 | | 318 | |  |
| C/G | 25 | | 29 | | 25 | |  |
| AC/GT | 2 | | 3 | | 2 | |  |
| AG/CT | 12 | | 11 | | 10 | |  |
| AT/AT | 28 | | 28 | | 27 | |  |
| CG/CG | 0 | | 1 | | 1 | |  |
| AAG/CTT | 1 | | 0 | | 1 | |  |
| AAT/ATT | 2 | | 3 | | 1 | |  |
| ATC/ATG | 1 | | 0 | | 0 | |  |
| ACT/AGT | 0 | | 0 | | 1 | |  |
| AAC/GTT | 0 | | 0 | | 1 | |  |
| AAAC/GTTT | | 0 | | 1 | | 1 | |
| AAAT/ATTT | | 2 | | 5 | | 4 | |
| AAGG/CCTT | | 0 | | 1 | | 0 | |
| AAGT/ACTT | | 0 | | 1 | | 0 | |
| AGGG/CCCT | | 0 | | 2 | | 2 | |
