# Supplementary material for: Intraspecific Variation within the Utricularia amethystina Species Morphotypes Based on Chloroplast Genomes
Source: Int J Mol Sci. 2019 Dec 5;20(24):6130. doi: 10.3390/ijms20246130 (PMC6940893; doi:10.3390/ijms20246130)
Supplement: Supplementary file 1 [file ijms-20-06130-s001.zip › Supplementary_Table_S6.docx]

| **Table S6**. Nucleotide diversity (π) between *Utricularia amethystina* cpDNAs. | |
| --- | --- |
| **End Position in chloroplast genome** | **Nucleotide diversity (π)** |
| 0 | - |
| 499 | 0.25267 |
| 999 | 0.004 |
| 1499 | 0.008 |
| 1999 | 0.04333 |
| 2499 | 0.02667 |
| 2999 | 0.03867 |
| 3499 | 0.04067 |
| 3999 | 0.046 |
| 4499 | 0.07 |
| 4999 | 0.12733 |
| 5499 | 0.07067 |
| 5999 | 0.098 |
| 6499 | 0.05333 |
| 6999 | 0.20733 |
| 7499 | 0.094 |
| 7999 | 0.052 |
| 8499 | 0.02333 |
| 8999 | 0.192 |
| 9499 | 0.032 |
| 9999 | 0.09667 |
| 10499 | 0.024 |
| 10999 | 0.00667 |
| 11499 | 0.008 |
| 11999 | 0.012 |
| 12499 | 0.038 |
| 12999 | 0.01867 |
| 13499 | 0.04133 |
| 13999 | 0.06267 |
| 14499 | 0.13 |
| 14999 | 0.06867 |
| 15499 | 0.00933 |
| 15999 | 0.054 |
| 16499 | 0.02 |
| 16999 | 0.062 |
| 17499 | 0.01067 |
| 17999 | 0.024 |
| 18499 | 0.02 |
| 18999 | 0.016 |
| 19499 | 0.032 |
| 19999 | 0.01867 |
| 20499 | 0.00933 |
| 20999 | 0.01867 |
| 21499 | 0.03333 |
| 21999 | 0.00533 |
| 22499 | 0.008 |
| 22999 | 0.052 |
| 23499 | 0.01733 |
| 23999 | 0.00533 |
| 24499 | 0.00667 |
| 24999 | 0.01067 |
| 25499 | 0.01067 |
| 25999 | 0.00667 |
| 26499 | 0.00533 |
| 26999 | 0.01 |
| 27499 | 0.07333 |
| 27999 | 0.07867 |
| 28499 | 0.068 |
| 28999 | 0.342 |
| 29499 | 0.412 |
| 29999 | 0.43933 |
| 30499 | 0.45 |
| 30999 | 0.291 |
| 31499 | 0.054 |
| 31999 | 0.11267 |
| 32499 | 0.09933 |
| 32999 | 0.162 |
| 33499 | 0.04267 |
| 33999 | 0.00933 |
| 34499 | 0.00667 |
| 34999 | 0.00533 |
| 35499 | 0.004 |
| 35999 | 0.092 |
| 36499 | 0.058 |
| 36999 | 0.11667 |
| 37499 | 0.06733 |
| 37999 | 0.02 |
| 38499 | 0.00667 |
| 38999 | 0.008 |
| 39499 | 0.008 |
| 39999 | 0.01 |
| 40499 | 0.00667 |
| 40999 | 0.00867 |
| 41499 | 0.00133 |
| 41999 | 0.004 |
| 42499 | 0.01867 |
| 42999 | 0.202 |
| 43499 | 0.07667 |
| 43999 | 0.038 |
| 44499 | 0.02733 |
| 44999 | 0.02267 |
| 45499 | 0.05 |
| 45999 | 0.34733 |
| 46499 | 0.082 |
| 46999 | 0.014 |
| 47499 | 0.07267 |
| 47999 | 0.056 |
| 48499 | 0.07533 |
| 48999 | 0.03467 |
| 49499 | 0.054 |
| 49999 | 0.07867 |
| 50499 | 0.016 |
| 50999 | 0.02133 |
| 51499 | 0.088 |
| 51999 | 0.12933 |
| 52499 | 0.01733 |
| 52999 | 0.04267 |
| 53499 | 0.03733 |
| 53999 | 0.00933 |
| 54499 | 0.012 |
| 54999 | 0.00933 |
| 55499 | 0.10267 |
| 55999 | 0.048 |
| 56499 | 0.016 |
| 56999 | 0.02267 |
| 57499 | 0.04267 |
| 57999 | 0.056 |
| 58499 | 0.04933 |
| 58999 | 0.026 |
| 59499 | 0.01067 |
| 59999 | 0.06467 |
| 60499 | 0.04333 |
| 60999 | 0.04533 |
| 61499 | 0.234 |
| 61999 | 0.15333 |
| 62499 | 0.048 |
| 62999 | 0.028 |
| 63499 | 0.00933 |
| 63999 | 0.07733 |
| 64499 | 0.02933 |
| 64999 | 0.00533 |
| 65499 | 0.004 |
| 65999 | 0.11133 |
| 66499 | 0.16667 |
| 66999 | 0.054 |
| 67499 | 0.076 |
| 67999 | 0.056 |
| 68499 | 0.02667 |
| 68999 | 0.06133 |
| 69499 | 0.00867 |
| 69999 | 0.08267 |
| 70499 | 0.028 |
| 70999 | 0.05 |
| 71499 | 0.04 |
| 71999 | 0.06067 |
| 72499 | 0.02533 |
| 72999 | 0.086 |
| 73499 | 0.012 |
| 73999 | 0.01467 |
| 74499 | 0.01333 |
| 74999 | 0.07867 |
| 75499 | 0.03733 |
| 75999 | 0.08533 |
| 76499 | 0.03 |
| 76999 | 0.032 |
| 77499 | 0.12933 |
| 77999 | 0.068 |
| 78499 | 0.05267 |
| 78999 | 0.032 |
| 79499 | 0.016 |
| 79999 | 0.03 |
| 80499 | 0.03867 |
| 80999 | 0.024 |
| 81499 | 0.02267 |
| 81999 | 0.032 |
| 82499 | 0.02933 |
| 82999 | 0.03933 |
| 83499 | 0.09667 |
| 83999 | 0.04733 |
| 84499 | 0.05333 |
| 84999 | 0.01 |
| 85499 | 0 |
| 85999 | 0.00267 |
| 86499 | 0.00133 |
| 86999 | 0.00267 |
| 87499 | 0 |
| 87999 | 0.008 |
| 88499 | 0.05867 |
| 88999 | 0.00133 |
| 89499 | 0.004 |
| 89999 | 0.00133 |
| 90499 | 0.004 |
| 90999 | 0.00133 |
| 91499 | 0.00667 |
| 91999 | 0.00333 |
| 92499 | 0.00267 |
| 92999 | 0.00133 |
| 93499 | 0.01733 |
| 93999 | 0.004 |
| 94499 | 0 |
| 94999 | 0.00133 |
| 95499 | 0 |
| 95999 | 0.008 |
| 96499 | 0.00533 |
| 96999 | 0.02333 |
| 97499 | 0.00267 |
| 97999 | 0.012 |
| 98499 | 0.00267 |
| 98999 | 0.01733 |
| 99499 | 0.01467 |
| 99999 | 0.01333 |
| 100499 | 0.004 |
| 100999 | 0 |
| 101499 | 0 |
| 101999 | 0 |
| 102499 | 0.00133 |
| 102999 | 0.00267 |
| 103499 | 0.00133 |
| 103999 | 0.00533 |
| 104499 | 0.03467 |
| 104999 | 0.00133 |
| 105499 | 0.00267 |
| 105999 | 0 |
| 106499 | 0.00133 |
| 106999 | 0 |
| 107499 | 0.00133 |
| 107999 | 0.04267 |
| 108499 | 0.01333 |
| 108999 | 0.01867 |
| 109499 | 0.01467 |
| 109999 | 0.008 |
| 110499 | 0.05267 |
| 110999 | 0.05667 |
| 111499 | 0.01867 |
| 111999 | 0.02267 |
| 112499 | 0.06 |
| 112999 | 0.14933 |
| 113499 | 0.24 |
| 113999 | 0.03533 |
| 114499 | 0.04867 |
| 114999 | 0.08 |
| 115499 | 0.026 |
| 115999 | 0.01333 |
| 116499 | 0.038 |
| 116999 | 0.07267 |
| 117499 | 0.05867 |
| 117999 | 0.024 |
| 118499 | 0.06733 |
| 118999 | 0.032 |
| 119499 | 0.02667 |
| 119999 | 0.088 |
| 120499 | 0.10667 |
| 120999 | 0.01067 |
| 121499 | 0.018 |
| 121999 | 0.028 |
| 122499 | 0.066 |
| 122999 | 0.05333 |
| 123499 | 0.12133 |
| 123999 | 0.024 |
| 124499 | 0.092 |
| 124999 | 0.06867 |
| 125499 | 0.06267 |
| 125999 | 0.03267 |
| 126499 | 0.05467 |
| 126999 | 0.07667 |
| 127499 | 0.052 |
| 127999 | 0.00667 |
| 128499 | 0.01733 |
| 128999 | 0.02533 |
| 129499 | 0.01733 |
| 129999 | 0.02133 |
