# Supplementary material for: Intraspecific Variation within the Utricularia amethystina Species Morphotypes Based on Chloroplast Genomes
Source: Int J Mol Sci. 2019 Dec 5;20(24):6130. doi: 10.3390/ijms20246130 (PMC6940893; doi:10.3390/ijms20246130)
Supplement: Supplementary file 1 [file ijms-20-06130-s001.zip › Supplementary_Table_S7.docx]

**Table S7.** Chloroplasts genes expression level of each *Utricularia amethystina* morphotype biological replicate. The underscore “_1” and “_2” denotes each gene duplicate. The *rps*12 is duplicated trans-spliced gene, therefore was analyzed in three parts.

| **Genes/ Species morphotypes** | ***U. amethystina***  **purple** | | | ***U. amethystina***  **white** | | | ***U. amethystina* yellow** | |
| --- | --- | --- | --- | --- | --- | --- | --- | --- |
|  | **P1** | **P2** | **P3** | **W1** | **W2** | **W3** | **Y1** | **Y2** |
| *acc*D | 60.31 | 63.99 | 43.1 | 40.03 | 49.83 | 44.01 | 78.86 | 111.96 |
| *atp*A | 16.24 | 13.42 | 18.2 | 74.77 | 85.82 | 45.08 | 81.14 | 73.82 |
| *atp*B | 37.11 | 45.41 | 41.18 | 74.01 | 78.9 | 39.18 | 36.57 | 36.91 |
| *atp*E | 8.12 | 8.26 | 11.49 | 16.62 | 24.92 | 7.51 | 10.29 | 13.53 |
| *atp*F | 0 | 0 | 0 | 26.43 | 12.46 | 15.03 | 41.14 | 31.99 |
| *atp*H | 15.08 | 17.55 | 16.28 | 14.35 | 18 | 13.95 | 24 | 18.46 |
| *atp*I | 35.95 | 30.96 | 36.39 | 39.27 | 48.45 | 48.3 | 34.29 | 34.45 |
| *ccs*A | 34.79 | 18.58 | 23.94 | 9.06 | 8.31 | 13.42 | 58.29 | 55.37 |
| *cem*A | 27.83 | 17.55 | 31.6 | 15.86 | 8.31 | 19.32 | 18.29 | 15.99 |
| *clp*P | 61.47 | 66.05 | 74.7 | 52.87 | 70.6 | 43.47 | 76.57 | 86.12 |
| *inf*A | 8.12 | 9.29 | 3.83 | 3.78 | 5.54 | 4.83 | 2.29 | 7.38 |
| *mat*K | 57.99 | 51.6 | 51.72 | 50.6 | 83.06 | 83.73 | 60.57 | 72.59 |
| *ndh*A | 26.68 | 19.61 | 17.24 | 15.86 | 13.84 | 30.59 | 19.43 | 18.46 |
| *ndh*B_1 | 8.12 | 4.13 | 3.83 | 13.59 | 11.07 | 10.2 | 4.57 | 2.46 |
| *ndh*B_2 | 0 | 0 | 0 | 7.55 | 11.07 | 0 | 0 | 0 |
| *ndh*C | 6.96 | 5.16 | 8.62 | 2.27 | 5.54 | 9.66 | 4.57 | 2.46 |
| *ndh*D | 61.47 | 40.25 | 52.67 | 49.85 | 45.68 | 46.16 | 48 | 38.14 |
| *ndh*E | 9.28 | 10.32 | 8.62 | 11.33 | 9.69 | 12.34 | 19.43 | 11.07 |
| *ndh*F | 17.4 | 12.39 | 18.2 | 6.8 | 12.46 | 18.78 | 10.29 | 9.84 |
| *ndh*G | 15.08 | 18.58 | 10.53 | 26.43 | 16.61 | 14.49 | 13.71 | 19.69 |
| *ndh*H | 22.04 | 17.55 | 11.49 | 12.08 | 12.46 | 18.78 | 11.43 | 8.61 |
| *ndh*I | 6.96 | 12.39 | 2.87 | 13.59 | 5.54 | 13.95 | 10.29 | 18.46 |
| *ndh*J | 5.8 | 4.13 | 13.41 | 4.53 | 5.54 | 7.51 | 2.29 | 2.46 |
| *ndh*K | 17.4 | 12.39 | 15.32 | 5.29 | 11.07 | 8.05 | 3.43 | 6.15 |
| *pet*A | 30.15 | 35.09 | 38.31 | 37.01 | 29.07 | 24.15 | 40 | 39.37 |
| *pet*B | 118.3 | 107.34 | 117.8 | 120.08 | 99.67 | 85.87 | 98.29 | 102.12 |
| *pet*D | 90.46 | 66.05 | 94.81 | 95.92 | 85.82 | 55.82 | 59.43 | 77.51 |
| *pet*G | 8.12 | 10.32 | 8.62 | 3.78 | 8.31 | 5.9 | 8 | 6.15 |
| *pet*L | 5.8 | 5.16 | 0 | 3.02 | 6.92 | 6.44 | 6.86 | 8.61 |
| *pet*N | 2.32 | 1.03 | 3.83 | 10.57 | 8.31 | 4.29 | 4.57 | 6.15 |
| *psa*A | 199.48 | 188.87 | 188.67 | 217.51 | 258.86 | 286.06 | 200 | 201.78 |
| *psa*B | 228.48 | 222.93 | 228.89 | 248.47 | 246.4 | 312.36 | 282.28 | 241.15 |
| *psa*C | 13.92 | 9.29 | 16.28 | 18.88 | 23.53 | 15.56 | 17.14 | 14.76 |
| *psa*I | 6.96 | 9.29 | 5.75 | 15.1 | 12.46 | 8.59 | 19.43 | 17.22 |
| *psa*J | 6.96 | 21.67 | 18.2 | 8.31 | 9.69 | 19.86 | 21.71 | 36.91 |
| *psb*A | 2420.48 | 2727.8 | 2735.21 | 3628.93 | 3772.14 | 3485.88 | 3414.84 | 3381.02 |
| *psb*B | 191.37 | 209.51 | 202.08 | 139.72 | 139.81 | 167.99 | 140.57 | 150.1 |
| *psb*C | 329.38 | 322.01 | 332.32 | 508.28 | 523.26 | 374.08 | 465.14 | 431.85 |
| *psb*D | 267.91 | 237.38 | 271.03 | 404.05 | 355.76 | 272.64 | 350.85 | 295.29 |
| *psb*E | 16.24 | 17.55 | 19.15 | 18.13 | 16.61 | 19.86 | 22.86 | 33.22 |
| *psb*F | 1.16 | 4.13 | 2.87 | 1.51 | 2.77 | 0.54 | 0 | 0 |
| *psb*H | 22.04 | 10.32 | 12.45 | 10.57 | 13.84 | 10.2 | 5.71 | 6.15 |
| *psb*I | 3.48 | 3.1 | 3.83 | 3.78 | 2.77 | 12.34 | 12.57 | 11.07 |
| *psb*J | 9.28 | 9.29 | 17.24 | 17.37 | 16.61 | 19.86 | 12.57 | 19.69 |
| *psb*K | 15.08 | 9.29 | 12.45 | 18.13 | 15.23 | 22.54 | 20.57 | 31.99 |
| *psb*L | 3.48 | 7.22 | 6.7 | 12.84 | 12.46 | 10.73 | 5.71 | 6.15 |
| *psb*M | 8.12 | 16.51 | 10.53 | 9.82 | 15.23 | 10.2 | 5.71 | 1.23 |
| *psb*N | 6.96 | 12.39 | 14.37 | 9.06 | 12.46 | 6.98 | 10.29 | 13.53 |
| *psb*T | 15.08 | 10.32 | 22.03 | 12.84 | 22.15 | 24.15 | 18.29 | 17.22 |
| *psb*Z | 51.03 | 43.35 | 50.76 | 89.12 | 83.06 | 60.11 | 77.71 | 83.66 |
| *rbc*L | 519.59 | 477.86 | 491.3 | 320.98 | 304.54 | 708.45 | 208 | 185.78 |
| *rpl*2_1 | 1.16 | 2.06 | 3.83 | 4.53 | 1.38 | 0.54 | 2.29 | 1.23 |
| *rpl*2_2 | 2.32 | 0 | 0 | 3.02 | 6.92 | 4.83 | 0 | 0 |
| *rpl*14 | 16.24 | 12.39 | 14.37 | 27.19 | 27.69 | 28.98 | 10.29 | 18.46 |
| *rpl*16 | 24.36 | 39.22 | 21.07 | 30.96 | 13.84 | 17.17 | 13.71 | 8.61 |
| *rpl*20 | 5.8 | 6.19 | 6.7 | 2.27 | 1.38 | 2.68 | 5.71 | 3.69 |
| *rpl*22 | 4.64 | 5.16 | 5.75 | 3.02 | 0 | 1.07 | 2.29 | 4.92 |
| *rpl*23_1 | 1.16 | 0 | 0 | 2.27 | 1.38 | 1.07 | 0 | 0 |
| *rpl*23_2 | 0 | 0 | 0 | 0 | 0 | 0 | 0 | 0 |
| *rpl*32 | 5.8 | 8.26 | 6.7 | 2.27 | 1.38 | 3.22 | 2.29 | 1.23 |
| *rpl*33 | 3.48 | 10.32 | 8.62 | 11.33 | 12.46 | 11.27 | 8 | 13.53 |
| *rpl*36 | 4.64 | 3.1 | 5.75 | 0.76 | 0 | 2.15 | 0 | 2.46 |
| *rpo*A | 24.36 | 17.55 | 23.94 | 17.37 | 18 | 32.2 | 20.57 | 18.46 |
| *rpo*B | 41.75 | 38.19 | 34.48 | 20.39 | 33.22 | 29.52 | 24 | 25.84 |
| *rpo*C1 | 28.99 | 10.32 | 13.41 | 20.39 | 8.31 | 16.1 | 18.29 | 11.07 |
| *rpo*C2 | 52.19 | 44.38 | 38.31 | 28.7 | 42.91 | 46.16 | 42.29 | 50.44 |
| *rps*11 | 12.76 | 10.32 | 19.15 | 11.33 | 6.92 | 12.34 | 8 | 11.07 |
| *rps*12_1 | 9.28 | 6.19 | 9.58 | 3.78 | 4.15 | 5.9 | 8 | 3.69 |
| *rps*12_2 | 0 | 0 | 0 | 2.27 | 1.38 | 0 | 1.14 | 1.23 |
| *rps*12_3 | 6.96 | 5.16 | 6.7 | 0.76 | 0 | 0.54 | 0 | 0 |
| *rps*14 | 47.55 | 37.16 | 25.86 | 24.92 | 34.61 | 25.22 | 48 | 33.22 |
| *rps*15 | 1.16 | 2.06 | 2.87 | 0 | 2.77 | 2.15 | 6.86 | 6.15 |
| *rps*16 | 32.47 | 35.09 | 27.77 | 25.68 | 27.69 | 15.56 | 38.86 | 40.6 |
| *rps*18 | 15.08 | 14.45 | 12.45 | 13.59 | 13.84 | 20.93 | 13.71 | 4.92 |
| *rps*19_1 | 1.16 | 2.06 | 3.83 | 1.51 | 0 | 0 | 2.29 | 1.23 |
| *rps*19_2 | 4.64 | 6.19 | 8.62 | 5.29 | 2.77 | 4.29 | 5.71 | 2.46 |
| *rps*2 | 16.24 | 14.45 | 14.37 | 12.08 | 12.46 | 13.42 | 11.43 | 17.22 |
| *rps*3 | 12.76 | 14.45 | 21.07 | 21.9 | 22.15 | 20.93 | 6.86 | 25.84 |
| *rps*4 | 11.6 | 6.19 | 7.66 | 3.78 | 5.54 | 5.9 | 88 | 109.5 |
| *rps*7_1 | 5.8 | 6.19 | 10.53 | 6.8 | 8.31 | 0.54 | 1.14 | 8.61 |
| *rps*7_2 | 0 | 0 | 0 | 3.78 | 5.54 | 3.76 | 0 | 0 |
| *rps*8 | 9.28 | 13.42 | 11.49 | 7.55 | 2.77 | 8.05 | 2.29 | 6.15 |
| *ycf*1 | 13.92 | 23.74 | 21.07 | 30.21 | 19.38 | 26.3 | 44.57 | 44.29 |
| *ycf*15_1 | 0 | 1.03 | 0 | 0 | 0 | 0 | 0 | 0 |
| *ycf*15_2 | 0 | 0 | 0 | 0.76 | 2.77 | 1.61 | 0 | 0 |
| *ycf*2_1 | 6.96 | 7.22 | 6.7 | 3.78 | 1.38 | 2.15 | 17.14 | 19.69 |
| *ycf*2_2 | 0 | 0 | 0 | 21.9 | 23.53 | 19.86 | 0 | 0 |
| *ycf*3 | 32.47 | 38.19 | 25.86 | 63.44 | 45.68 | 55.28 | 105.14 | 105.81 |
| *ycf*4 | 39.43 | 34.06 | 36.39 | 25.68 | 27.69 | 15.03 | 29.71 | 36.91 |
| trnA-UGC_1 | 0 | 3.1 | 5.75 | 0 | 0 | 0 | 0 | 3.69 |
| trnA-UGC_2 | 0 | 0 | 0 | 9.82 | 15.23 | 4.83 | 0 | 0 |
| trnC-GCA | 1.16 | 1.03 | 1.92 | 0.76 | 0 | 1.07 | 1.14 | 2.46 |
| trnD-GUC | 0 | 1.03 | 0 | 0.76 | 0 | 0 | 0 | 0 |
| trnE-UUC | 0 | 0 | 0 | 0 | 0 | 0 | 0 | 0 |
| trnF-GAA | 0 | 0 | 0 | 0 | 1.38 | 0 | 2.29 | 1.23 |
| trnfM-CAU | 0 | 0 | 0 | 0 | 0 | 0.54 | 0 | 0 |
| trnG-GCC | 0 | 0 | 0 | 0 | 0 | 0 | 0 | 0 |
| trnG-UCC | 19.72 | 15.48 | 19.15 | 0 | 0 | 0 | 19.43 | 29.53 |
| trnH-GUG | 4.64 | 6.19 | 11.49 | 3.78 | 1.38 | 7.51 | 6.86 | 3.69 |
| trnI-CAU_1 | 0 | 0 | 0 | 0 | 0 | 0 | 0 | 0 |
| trnI-CAU_2 | 0 | 0 | 0 | 0 | 0 | 0 | 0 | 0 |
| trnI-GAU_1 | 0 | 0 | 3.83 | 0 | 1.38 | 0 | 0 | 1.23 |
| trnI-GAU_2 | 0 | 0 | 0 | 14.35 | 24.92 | 14.49 | 0 | 0 |
| trnK-UUU | 2.32 | 8.26 | 4.79 | 12.84 | 16.61 | 11.27 | 11.43 | 3.69 |
| trnL-CAA_1 | 0 | 0 | 0 | 0 | 0 | 0 | 0 | 0 |
| trnL-CAA_2 | 0 | 0 | 0 | 0 | 0 | 0 | 0 | 0 |
| trnL-UAA | 1.16 | 3.1 | 0.96 | 1.51 | 2.77 | 2.68 | 3.43 | 6.15 |
| trnL-UAG | 1.16 | 0 | 0 | 0 | 0 | 0 | 0 | 1.23 |
| trnM-CAU | 6.96 | 2.06 | 0.96 | 0 | 1.38 | 0 | 1.14 | 1.23 |
| trnN-GUU_1 | 0 | 0 | 0 | 0 | 0 | 0 | 0 | 1.23 |
| trnN-GUU_2 | 0 | 0 | 0 | 0 | 0 | 0 | 0 | 0 |
| trnP-UGG | 6.96 | 7.22 | 11.49 | 1.51 | 2.77 | 8.05 | 6.86 | 19.69 |
| trnQ-UUG | 1.16 | 1.03 | 0 | 2.27 | 1.38 | 1.07 | 0 | 1.23 |
| trnR-ACG_1 | 0 | 0 | 0 | 0 | 0 | 0 | 0 | 0 |
| trnR-ACG_2 | 0 | 0 | 0 | 1.51 | 0 | 0 | 0 | 0 |
| trnR-UCU | 4.64 | 8.26 | 8.62 | 5.29 | 2.77 | 0.54 | 2.29 | 9.84 |
| trnS-GCU | 1.16 | 3.1 | 0.96 | 46.07 | 51.22 | 62.79 | 5.71 | 6.15 |
| trnS-GGA | 2.32 | 2.06 | 5.75 | 3.02 | 1.38 | 0 | 17.14 | 13.53 |
| trnS-UGA | 54.51 | 48.51 | 33.52 | 32.48 | 30.45 | 44.55 | 85.71 | 83.66 |
| trnT-GGU | 0 | 0 | 0 | 0 | 0 | 0 | 0 | 1.23 |
| trnT-UGU | 0 | 0 | 0 | 0.76 | 0 | 0 | 0 | 0 |
| trnV-GAC_1 | 0 | 0 | 0 | 0 | 0 | 0 | 0 | 0 |
| trnV-GAC_2 | 0 | 0 | 0 | 0 | 0 | 0 | 0 | 0 |
| trnV-UAC | 2.32 | 3.1 | 1.92 | 10.57 | 13.84 | 5.37 | 11.43 | 6.15 |
| trnW-CCA | 12.76 | 7.22 | 6.7 | 4.53 | 2.77 | 5.37 | 3.43 | 7.38 |
| trnY-GUA | 0 | 0 | 0 | 0 | 0 | 0 | 0 | 0 |
| 16S rRNA_1 | 1486.85 | 1495.49 | 2159.63 | 459.19 | 357.14 | 67.09 | 2286.84 | 2356.13 |
| 16S rRNA_2 | 2.32 | 5.16 | 13.41 | 3769.4 | 2967.88 | 556.02 | 9.14 | 13.53 |
| 23S rRNA_1 | 2579.38 | 2552.35 | 3668.02 | 27.94 | 19.38 | 2.68 | 4028.55 | 4007.27 |
| 23S rRNA_2 | 27.83 | 25.8 | 25.86 | 6078.93 | 4774.36 | 1111.51 | 28.57 | 46.75 |
| 4.5S rRNA_1 | 10.44 | 17.55 | 13.41 | 15.1 | 12.46 | 5.37 | 2.29 | 11.07 |
| 4.5S rRNA_2 | 0 | 0 | 0 | 30.96 | 16.61 | 5.9 | 0 | 0 |
| 5S rRNA_1 | 0 | 2.06 | 0.96 | 0 | 0 | 2.15 | 2.29 | 3.69 |
| 5S rRNA_2 | 0 | 0 | 0 | 1.51 | 4.15 | 0 | 0 | 0 |
