# Supplementary material for: Intraspecific Variation within the Utricularia amethystina Species Morphotypes Based on Chloroplast Genomes
Source: Int J Mol Sci. 2019 Dec 5;20(24):6130. doi: 10.3390/ijms20246130 (PMC6940893; doi:10.3390/ijms20246130)
Supplement: Supplementary file 1 [file ijms-20-06130-s001.zip › Supplementary_Table_S8-S10.docx]

**Table S8.** RNA editing sites in *Utricularia amethystina* purple cpDNA predicted by PREPACT**3**. * denotes for amino acids with changes in its physico-chemical composition.

| **Gene** | **Genome (cp)** | | **Codon** | | **Codon position** | **Amino acid** | |
| --- | --- | --- | --- | --- | --- | --- | --- |
|  | **Position** | **Strand** | **from** | **to** |  | **from** | **to** |
| *acc*D | 56676 | + | ACG | AUG | 2 | T | M |
|  | 56739 | + | GCU | GUU | 2 | A | V |
|  | 56786 | + | CAU | UAU | 1 | H | Y |
|  | 56808 | + | ACU | AUU | 2 | T | I |
|  | 56918 | + | CCU | UCU | 1 | P | S |
|  | 56985* | + | UCU | UUU | 2 | S | F |
|  | 57108 | + | ACU | AUU | 2 | T | I |
|  | 57396* | + | UCG | UUG | 2 | S | L |
|  | 57774* | + | UCA | UUA | 2 | S | L |
| *atp*I | 15119 | - | CCU | CUU | 2 | P | L |
|  | 14522* | - | UCA | UUA | 2 | S | L |
| *ccs*A | 11304 | + | CUU | UUU | 1 | L | F |
|  | 111311 | + | GCC | GUC | 2 | A | V |
|  | 111710 | + | GCC | GUC | 2 | A | V |
| *mat*K | 3297 | - | CCU | UCU | 1 | P | S |
|  | 3294 | - | CUU | UUU | 1 | L | F |
|  | 3092 | - | ACA | AUA | 2 | T | I |
|  | 2895 | - | CAU | UAU | 1 | H | Y |
|  | 2741 | - | GCG | GUG | 2 | A | V |
|  | 2660* | - | UCC | UUC | 2 | S | F |
|  | 2294* | - | UCU | UUU | 2 | S | F |
|  | 2286 | - | CAU | UAU | 1 | H | Y |
|  | 2091 | - | CCA | UCA | 1 | P | S |
| *ndh*A | 118328* | - | UCA | UUA | 2 | S | L |
| *ndh*B | 137581* | - | UCA | UUA | 2 | S | L |
|  | 138169 | - | CCA | CUA | 2 | P | L |
|  | 138288 | - | CAU | UAU | 1 | H | Y |
|  | 138439 | - | CCA | CUA | 2 | P | L |
|  | 138448* | - | UCU | UUU | 2 | S | F |
|  | 139276* | - | UCA | UUA | 2 | S | L |
|  | 139222* | - | UCA | UUA | 2 | S | L |
|  | 139678* | - | UCC | UUC | 2 | S | F |
| *ndh*D | 113897 | - | ACG | AUG | 2 | T | M |
|  | 113892 | - | CAU | UAU | 1 | H | Y |
|  | 113873 | - | ACA | AUA | 2 | T | I |
|  | 113774* | - | UCG | UUG | 2 | S | L |
|  | 113297 | - | GCC | GUC | 2 | A | V |
|  | 113276 | - | GCC | GUC | 2 | A | V |
|  | 113021* | - | UCA | UUA | 2 | S | L |
|  | 112641 | - | CUU | UUU | 1 | L | F |
|  | 112439 | - | GCU | GUU | 2 | A | V |
|  | 112409* | - | UCU | UUU | 2 | S | F |
| *ndh*F | 109880 | - | CUU | UUU | 1 | L | F |
|  | 109807 | - | GCC | GUC | 2 | A | V |
|  | 109282 | - | GCU | GUU | 2 | A | V |
|  | 108787 | - | GCA | GUA | 2 | A | V |
|  | 108668 | - | CUC | UUC | 1 | L | F |
|  | 108485* | - | CGU | UGU | 1 | R | C |
|  | 108284 | - | CUU | UUU | 1 | L | F |
|  | 107803 | - | GCA | GUA | 2 | A | V |
|  | 107791 | - | ACA | AUA | 2 | T | I |
| *ndh*G | 115562 | - | CCU | UCU | 1 | P | S |
|  | 115534 | - | GCA | GUA | 2 | A | V |
|  | 115258 | - | ACA | AUA | 2 | T | I |
| *pet*B | 74797 | + | CCA | CUA | 2 | P | L |
| *psa*I | 58577 | + | CCU | UCU | 1 | P | S |
| *psb*B | 71381* | + | CGU | UGU | 1 | R | C |
|  | 71454 | + | GCG | GUG | 2 | A | V |
| *rpl*2 | 149637 | + | GCC | GUG | 2 | A | V |
| *rpl*20 | 67150* | + | UCA | UUA | 2 | S | L |
| *rpo*A | 77209* | - | UCU | UUU | 2 | S | F |
|  | 76916 | - | CUU | UUU | 1 | L | F |
|  | 76879 | - | ACG | AUG | 2 | T | M |
|  | 76594* | - | UCA | UUA | 2 | S | L |
|  | 76583 | - | CCU | UCU | 1 | P | S |
|  | 76484 | - | CCA | UCA | 1 | P | S |
|  | 76445 | - | CAU | UAU | 1 | H | Y |
| *rpo*B | 26588 | - | CUU | UUU | 1 | L | F |
|  | 26080* | - | UCA | UUA | 2 | S | L |
|  | 25714 | - | ACG | AUG | 2 | T | M |
|  | 25093 | - | GCC | GUC | 2 | A | V |
|  | 24823 | - | ACU | AUU | 2 | T | I |
|  | 24760 | - | CCU | CUU | 2 | P | L |
|  | 24205* | - | UCA | UUA | 2 | S | L |
|  | 24092 | - | CCC | UCC | 1 | P | S |
| *rpoC1* | 20759 | - | ACC | AUC | 2 | T | I |
| *rpoC2* | 20035 | - | CUU | UUU | 1 | L | F |
|  | 19576 | - | CUU | UUU | 1 | L | F |
|  | 18903* | - | UCU | UUU | 2 | S | F |
|  | 18832 | - | CCU | UCU | 1 | P | S |
|  | 18742 | - | CCG | UCG | 1 | P | S |
|  | 18531 | - | ACU | AUU | 2 | T | I |
|  | 18424 | - | CUU | UUU | 1 | L | F |
|  | 18256 | - | CUU | UUU | 1 | L | F |
|  | 17662 | - | CCC | UCC | 1 | P | S |
|  | 16698* | - | UCA | UUA | 2 | S | L |
|  | 16623 | - | GCA | GUA | 2 | A | V |
| *rps*2 | 16076 | - | ACU | AUU | 2 | T | I |
|  | 15965 | - | ACA | AUA | 2 | T | I |
|  | 15851* | - | UCA | UUA | 2 | S | L |
|  | 15848 | - | GCA | GUA | 2 | A | V |
|  | 15843 | - | CGG | UGG | 1 | R | W |
|  | 15390 | - | CAA | UAA | 1 | Q | STOP |
| *rps*14 | 36640* | - | UCA | UUA | 2 | S | L |
| *psb*K | 7392 | + | CUU | UUU | 1 | L | F |
| *rbc*L | 55680 | + | GCA | GUA | 2 | A | V |
| *ycf*4 | 59210 | + | CUU | UUU | 1 | L | F |
| *cem*A | 60048 | + | CUU | UUU | 1 | L | F |
| *rpl*33 | 66183 | + | GCG | GUG | 2 | A | V |
|  | 66195 | + | GCG | GUG | 2 | A | V |
| *rps*18 | 66552* | + | UCC | UUC | 2 | S | F |
|  | 66803 | + | CUU | UUU | 1 | L | F |
| *psb*H | 73174 | + | GCA | GUA | 2 | A | V |
| *pet*B | 74797 | + | CCA | CUA | 2 | P | L |
| *rpl*14 | 79325* | - | CGU | UGU | 1 | R | C |
| *rpl*22 | 82134* | - | UCG | UUG | 2 | S | L |
|  | 81793 | - | CCU | UCU | 1 | P | S |
| *rpl*23 | 148159* | - | UCU | UUU | 2 | S | F |
|  | 148177* | - | UCA | UUA | 2 | S | L |
| *ycf*2 | 147162 | - | GCC | GUC | 2 | A | V |
|  | 146700 | - | GCG | GUG | 2 | A | V |
|  | 146302 | - | CUU | UUU | 1 | L | F |
|  | 145912 | - | CAC | UAC | 1 | H | Y |
|  | 145815* | - | UCU | UUU | 2 | S | F |
|  | 145393 | - | CAU | UAU | 1 | H | Y |
|  | 145263* | - | UCC | UUC | 2 | S | F |
|  | 144181 | - | CCA | UCA | 1 | P | S |
|  | 143452 | - | CCA | UCA | 1 | P | S |
|  | 143029 | - | CCC | UCC | 1 | P | S |
|  | 142230 | - | GCC | GUC | 2 | A | V |
|  | 141886 | - | CCC | UCC | 1 | P | S |
|  | 141702* | - | UCA | UUA | 2 | S | L |
|  | 141348 | - | CCU | CUU | 2 | P | L |
|  | 141306 | - | CCC | CUC | 2 | P | L |
| *rpl*32 | 110661 | + | CAA | UAA | 1 | Q | STOP |
| *ndh*E | 114578 | - | CCG | CUG | 2 | P | L |
| *ndh*I | 115848* | + | UCA | UUA | 2 | S | L |
| *ndh*H | 119347 | - | CAU | UAU | 1 | H | Y |
|  | 118977 | - | ACC | AUC | 2 | T | I |
| *rps*15 | 120103* | - | UCA | UUA | 2 | S | L |
| *ycf*1 | 124913* | - | UCA | UUA | 2 | S | L |
|  | 124800 | - | CCA | UCA | 1 | P | S |
|  | 124533 | - | CCC | UCC | 1 | P | S |
|  | 124460* | - | UCG | UUG | 2 | S | L |
|  | 124358 | - | ACU | AUU | 2 | T | I |
|  | 124275 | - | CAU | UAU | 1 | H | Y |
|  | 124155 | - | CUU | UUU | 1 | L | F |
|  | 123923 | - | ACU | AUU | 2 | T | I |
|  | 123614 | - | GCA | GUA | 2 | A | V |
|  | 123576  123575 | - | CCU | UUU | 1 and 2 | P | F |
|  | 123509 | - | ACA | AUA | 2 | T | I |
|  | 122915 | - | CCA | CUA | 2 | P | L |
|  | 122744 | - | ACA | AUA | 2 | T | I |
|  | 122664  122663 | - | CCU | UUU | 1 and 2 | P | F |
|  | 122129* | - | UCG | UUG | 2 | S | L |
|  | 121922 | - | ACC | AUC | 2 | T | I |
|  | 121886 | - | ACA | AUA | 2 | T | I |
|  | 121748 | - | CCC | CUC | 2 | P | L |
|  | 121743 | - | CUU | UUU | 1 | L | F |
|  | 120875* | - | UCA | UUA | 2 | S | L |

**Table S9.** RNA editing sites in *Utricularia amethystina* white cpDNA predicted by PREPACT**3**. * denotes for amino acids with changes in its physico-chemical composition.

| **Gene** | **Genome (cp)** | | **Codon** | | **Codon position** | **Amino acid** | |
| --- | --- | --- | --- | --- | --- | --- | --- |
|  | **Position** | **Strand** | **from** | **to** |  | **from** | **to** |
| *acc*D | 56613 | + | ACG | AUG | 2 | T | M |
|  | 56676 | + | GCG | GUG | 2 | A | V |
|  | 56723 | + | CAU | UAU | 1 | H | Y |
|  | 56745 | + | ACU | AUU | 2 | T | I |
|  | 56855 | + | CCU | UCU | 1 | P | S |
|  | 56922* | + | UCU | UUU | 2 | S | F |
|  | 57042 | + | ACU | AUU | 2 | T | I |
|  | 57324* | + | UCG | UUG | 2 | S | L |
|  | 57702* | + | UCA | UUA | 2 | S | L |
| *atp*I | 15055 | - | CCU | CUU | 2 | P | L |
|  | 14458* | - | UCA | UUA | 2 | S | L |
| *ccs*A | 111378 | + | CUU | UUU | 1 | L | F |
|  | 111385 | + | GCC | GUG | 2 | A | V |
|  | 111784 | + | GCC | GUG | 2 | A | V |
| *cem*A | 60111 | + | CUU | UUU | 1 | L | F |
| *mat*K | 3301 | - | CCU | UCU | 1 | P | S |
|  | 3096 | - | ACA | AUA | 2 | T | I |
|  | 2899 | - | CAU | UAU | 1 | H | Y |
|  | 2664* | - | UCU | UUU | 2 | S | F |
|  | 2298* | - | UCU | UUU | 2 | S | F |
|  | 2290 | - | CAU | UAU | 1 | H | Y |
|  | 2095 | - | CCA | UCA | 1 | P | S |
|  | 2034* | - | UCG | UUG | 2 | S | L |
| *ndh*A | 118308* | - | UCA | UUA | 2 | S | L |
| *ndh*B | 139170* | + | UCA | UUA | 2 | S | L |
|  | 139176* | + | UCA | UUA | 2 | S | L |
|  | 139632* | + | UCC | UUC | 2 | S | F |
|  | 139821 | + | CCA | CUA | 2 | P | L |
|  | 137802* | + | UCA | UUA | 2 | S | L |
|  | 138120 | + | CCA | CUA | 2 | P | L |
|  | 138239* | + | CAU | UAU | 1 | H | Y |
|  | 138390 | + | CCA | CUA | 2 | P | L |
|  | 138399* | + | UCU | UUU | 2 | S | F |
| *ndh*D | 113937 | - | ACG | AUG | 2 | T | M |
|  | 113932 | - | CAU | UAU | 1 | H | Y |
|  | 113913 | - | ACA | AUA | 2 | T | I |
|  | 113814* | - | UCG | UUG | 2 | S | L |
|  | 113337 | - | GCC | GUC | 2 | A | V |
|  | 113316 | - | GCC | GUC | 2 | A | V |
|  | 113061* | - | UCA | UUA | 2 | S | L |
|  | 112681 | - | CUU | UUU | 1 | L | F |
|  | 112479 | - | GCU | GUU | 2 | A | V |
|  | 112449* | - | UCU | UUU | 2 | S | F |
| *ndh*F | 109811 | - | CUU | UUU | 1 | L | F |
|  | 109738 | - | GCC | GUC | 2 | A | V |
|  | 109213 | - | GCU | GUU | 2 | A | V |
|  | 108718 | - | GCA | GUA | 2 | A | V |
|  | 108599 | - | CUC | UUC | 1 | L | F |
|  | 108215 | - | CUU | UUU | 1 | L | F |
|  | 107746 | - | GCA | GUA | 2 | A | V |
|  | 107734 | - | ACA | AUA | 2 | T | I |
| *ndh*G | 115603 | - | CCU | UCU | 1 | P | S |
|  | 115575 | - | GCA | GUA | 2 | A | V |
|  | 115299 | - | ACA | AUA | 2 | T | I |
| *pet*B | 74816 | + | CCA | CUA | 2 | P | L |
| *psa*I | 58490 | + | CCU | UCU | 1 | P | S |
| *psb*B | 71391* | + | CGU | UGU | 1 | R | C |
|  | 71464 | + | GCG | GUG | 2 | A | V |
| *psb*K | 7389 | - | CUU | UUU | 1 | L | F |
| *rpl*2 | 149540 | + | GCG | GUG | 2 | A | V |
| *rpl*20 | 67181* | - | UCA | UUA | 2 | S | L |
| *rpo*A | 77152* | - | UCU | UUU | 2 | S | F |
|  | 76859 | - | CUU | UUU | 1 | L | F |
|  | 76543* | - | UCA | UUA | 2 | S | L |
|  | 76532 | - | CCU | UCU | 1 | P | S |
|  | 76433 | - | CCA | UCA | 1 | P | S |
|  | 76394 | - | CAU | UAU | 1 | H | Y |
| *rpo*B | 26530 | - | CUU | UUU | 1 | L | F |
|  | 26022* | - | UCA | UUA | 2 | S | L |
|  | 25656 | - | ACG | AUG | 2 | T | M |
|  | 25035 | - | GCC | GUC | 2 | A | V |
|  | 24765 | - | ACU | AUU | 2 | T | I |
|  | 24702 | - | CCC | CUC | 2 | P | L |
|  | 24147* | - | UCA | UUA | 2 | S | L |
|  | 24034 | - | CCC | UCC | 1 | P | S |
| *rpoC1* | 20700 | - | ACC | AUC | 2 | T | I |
| *rpoC2* | 19976 | - | CUU | UUU | 1 | L | F |
|  | 19517 | - | CUU | UUU | 1 | L | F |
|  | 18844* | - | UCU | UUU | 2 | S | F |
|  | 18773 | - | CCU | UCU | 1 | P | S |
|  | 18683 | - | CCU | UCU | 1 | P | S |
|  | 18472 | - | ACU | AUU | 2 | T | I |
|  | 18365 | - | CUU | UUU | 1 | L | F |
|  | 18197 | - | CUU | UUU | 1 | L | F |
|  | 17603 | - | CCC | UCC | 1 | P | S |
|  | 17339 | - | CAU | UAU | 1 | H | Y |
|  | 16639* | - | UCA | UUA | 2 | S | L |
|  | 16564 | - | GCA | GUA | 2 | A | V |
| *rps*2 | 16034 | - | UCC | AUC | 2 | T | I |
|  | 15923 | - | ACA | AUA | 2 | T | I |
|  | 15809* | - | UCA | UUA | 2 | S | L |
|  | 15801 | - | CGG | UGG | 1 | R | W |
|  | 15348 | - | CAA | UAA | 1 | Q | STOP |
| *rps*14 | 36572* |  | UCA | UUA | 2 | S | L |
|  | 36497 |  | CCA | CUA | 2 | P | L |
| *rbc*L | 55635 | + | GCA | GUA | 2 | A | V |
| *ycf*4 | 59147 | + | CUU | UUU | 1 | L | F |
| *rpl*33 | 66220 | + | GCC | GUC | 2 | A | V |
|  | 66232 | + | GCG | GUG | 2 | A | V |
| *rps*18 | 66589* | + | UCC | UUC | 2 | S | F |
|  | 66849 | + | CUU | UUU | 1 | L | F |
| *psb*H | 73182 | + | GCA | GUA | 2 | A | V |
| *rpl*14 | 79295* | - | CGU | UGU | 1 | R | C |
| *rpl*22 | 82105* | - | UCA | UUA | 2 | S | L |
| *rpl23* | 84273* | - | UCU | UUU | 2 | S | F |
|  | 84255* | - | UCA | UUA | 2 | S | L |
| *ycf*2 | 147065 | - | GCC | GUC | 2 | A | V |
|  | 146244 | - | CUU | UUU | 1 | L | F |
|  | 145854 | - | CAC | UAC | 1 | H | Y |
|  | 145757* | - | UCU | UUU | 2 | S | F |
|  | 145335 | - | CAU | UAU | 1 | H | Y |
|  | 145205* | - | UCC | UUC | 2 | S | F |
|  | 144123 | - | CCA | UCA | 1 | P | S |
|  | 143400 | - | CCA | UCA | 1 | P | S |
|  | 142977 | - | CCC | UCC | 1 | P | S |
|  | 142178 | - | GCC | GUC | 2 | A | V |
|  | 141834 | - | CCC | UCC | 1 | P | S |
|  | 141650* | - | UCA | UUA | 2 | S | L |
|  | 141296 | - | CCU | CUU | 2 | P | L |
|  | 141254 | - | CCC | CUC | 2 | P | L |
| *rpl*32 | 110586 | + | CAA | UAA | 1 | Q | STOP |
| *ndh*E | 114594 | - | CCG | CUG | 2 | P | L |
| *ndh*I | 115871* | - | UCA | UUA | 2 | S | L |
| *ndh*H | 119524 | - | GCU | GUU | 2 | A | V |
|  | 119327 | - | CAU | UAU | 1 | H | Y |
|  | 118957 | - | ACC | AUC | 2 | T | I |
| *rps*15 | 120064* | - | UCA | UUA | 2 | S | L |
| *ycf*1 | 122717 | - | ACA | AUA | 2 | T | I |
|  | 122708* | - | UCU | UUU | 2 | S | F |
|  | 122436  122435 | - | CCU | UUU | 1  2 | P | F |
|  | 122250* | - | CGU | UGU | 1 | R | C |
|  | 122051* | - | UCG | UUG | 2 | S | L |
|  | 121844 | - | ACC | AUC | 2 | T | I |
|  | 121808 | - | ACA | AUA | 2 | T | I |
|  | 121737 | - | CCC | UCC | 1 | P | S |
|  | 121670 | - | CCC | CUC | 2 | P | L |
|  | 121665 | - | CUU | UUU | 1 | L | F |
|  | 120812* | - | UCA | UUA | 2 | S | L |

**Table S10.** RNA editing sites in *Utricularia amethystina* yellow cpDNA predicted by PREPACT**3**. * denotes for amino acids with changes in its physico-chemical composition.

| **Gene** | **Genome (cp)** | | **Codon** | | **Codon position** | **Amino acid** | |
| --- | --- | --- | --- | --- | --- | --- | --- |
|  | **Position** | **Strand** | **from** | **to** |  | **from** | **to** |
| *acc*D | 56465 | + | ACG | AUG | 2 | T | M |
|  | 56528 | + | GCU | GUU | 2 | A | V |
|  | 56575 | + | CAU | UAU | 1 | H | Y |
|  | 56597 | + | ACU | AUU | 2 | T | I |
|  | 56707 | + | CCU | UCU | 1 | P | S |
|  | 56774* | + | UCU | UUU | 1 | S | F |
|  | 56897 | + | ACU | AUU | 2 | T | I |
|  | 57185* | + | UCG | UUG | 2 | S | L |
|  | 57563* | + | UCA | UUA | 2 | S | L |
| *atp*I | 14940 | - | CCU | CUU | 2 | P | L |
|  | 14343* | - | UCA | UUA | 2 | S | L |
| *ccs*A | 111623 | + | CUU | UUU | 1 | L | F |
|  | 111630 | + | GCC | GUC | 2 | A | V |
|  | 112136 | + | CUU | UUU | 1 | L | F |
| *mat*K | 3182 | - | CCU | UCU | 1 | P | S |
|  | 3179 | - | CUU | UUU | 1 | L | F |
|  | 2977 | - | ACA | AUA | 2 | T | I |
|  | 2780 | - | CAU | UAU | 1 | H | Y |
|  | 2626 | - | GCG | GUG | 2 | A | V |
|  | 2611* | - | UCC | UUC | 2 | S | F |
|  | 2545* | - | UCU | UUU | 2 | S | F |
|  | 2179* | - | UCU | UUU | 2 | S | F |
|  | 2171 | - | CAU | UAU | 1 | H | Y |
|  | 1976 | - | CCA | UCA | 1 | P | S |
| *ndh*A | 812 | - | GCA | GUA | 2 | A | V |
| *ndh*B | 138105* | - | UCA | UUA | 2 | S | L |
|  | 138423 | - | CCA | CUA | 2 | P | L |
|  | 138542 | - | CAU | UAU | 1 | H | Y |
|  | 138693 | - | CCA | CUA | 2 | P | L |
|  | 138702* | - | UCU | UUU | 2 | S | F |
|  | 139473* | - | UCA | UUA | 2 | S | L |
|  | 139479* | - | UCA | UUA | 2 | S | L |
|  | 139935* | - | UCC | UUC | 2 | S | F |
|  | 140124 | - | CCA | CUA | 2 | P | L |
| *ndh*D | 114165 | - | ACG | AUG | 2 | T | M |
|  | 114160 | - | CAU | UAU | 1 | H | Y |
|  | 114141 | - | ACA | AUA | 2 | T | I |
|  | 113565 | - | GCC | GUC | 2 | A | V |
|  | 113544 | - | GCC | GUC | 2 | A | V |
|  | 113289* | - | UCA | UUA | 2 | S | L |
|  | 112909 | - | CUU | UUU | 1 | L | F |
|  | 112707 | - | GCU | GUU | 2 | A | V |
|  | 112677* | - | UCU | UUU | 2 | S | F |
| *ndh*F | 110043 | - | CUU | UUU | 1 | L | F |
|  | 109970 | - | GCC | GUC | 2 | A | V |
|  | 109445 | - | GCU | GUU | 2 | A | V |
|  | 108950 | - | GCA | GUA | 2 | A | V |
|  | 108831 | - | CUC | UUC | 1 | L | F |
|  | 108447 | - | CUU | UUU | 1 | L | F |
|  | 107966 | - | GCA | GUA | 2 | A | V |
|  | 107954 | - | ACA | AUA | 2 | T | I |
| *ndh*G | 115861 | - | CCU | UCU | 1 | P | S |
|  | 115557 | - | ACA | AUA | 2 | T | I |
| *pet*B | 74948 | + | CCA | CUA | 2 | P | L |
| *psa*I | 58347 | + | CCU | UCU | 1 | P | S |
| *psb*B | 71482* | + | CGU | UGU | 1 | R | C |
|  | 71555 | + | GCG | GUG | 2 | A | V |
| *rpl*2 | 149854 | + | GCG | GUG | 2 | A | V |
| *rpl*20 | 67248* | - | UCA | UUA | 2 | S | L |
| *rpo*A | 77400* | - | UCU | UUU | 2 | S | F |
|  | 77107 | - | CUU | UUU | 1 | L | F |
|  | 77070 | - | ACG | AUG | 2 | T | M |
|  | 76785* | - | UCA | UUA | 2 | S | L |
|  | 76774 | - | CCU | UCU | 1 | P | S |
|  | 76675 | - | CCA | UCA | 1 | P | S |
|  | 76636 | - | CAU | UAU | 1 | H | Y |
| *rpo*B | 26389 | - | CUU | UUU | 1 | L | F |
|  | 25881* | - | UCA | UUA | 2 | S | L |
|  | 25515 | - | ACG | AUG | 2 | T | M |
|  | 24894 | - | GCC | GUC | 2 | A | V |
|  | 24624 | - | ACU | AUU | 2 | T | I |
|  | 24561 | - | CCU | CUU | 2 | P | L |
|  | 24006* | - | UCA | UUA | 2 | S | L |
|  | 23893 | - | CCC | UCC | 1 | P | S |
| *rpoC1* | 20577 | - | ACC | AUC | 2 | T | I |
| *rpoC2* | 19853 | - | CUC | UUC | 1 | L | F |
|  | 19394 | - | CUU | UUU | 1 | L | F |
|  | 18721* | - | UCU | UUU | 2 | S | F |
|  | 18650 | - | CCU | UCU | 1 | P | S |
|  | 18560 | - | CCG | UCG | 1 | P | S |
|  | 18475 | - | CCC | CUC | 2 | P | L |
|  | 18349 | - | ACU | AUU | 2 | T | I |
|  | 18242 | - | CUU | UUU | 1 | L | F |
|  | 18074 | - | CUU | UUU | 1 | L | F |
|  | 17480 | - | CCC | UCC | 1 | P | S |
|  | 16516* | - | UCA | UUA | 2 | S | L |
|  | 16441 | - | GCA | GUA | 2 | A | V |
| *rps*2 | 15919 | - | ACU | AUU | 2 | T | I |
|  | 15808 | - | ACA | AUA | 2 | T | I |
|  | 15760 | - | GCU | GUU | 2 | A | V |
|  | 15694* | - | UCA | UUA | 2 | S | L |
|  | 15686 | - | CGG | UGG | 1 | R | W |
|  | 15233 | - | CAA | UAA | 1 | Q | STOP |
| *rps*14 | 36514* | - | UCA | UUA | 2 | S | L |
|  | 36439 | - | CCA | CUA | 2 | P | L |
| *psb*K | 7229 | + | CUU | UUU | 1 | L | F |
| *rbc*L | 55485 | + | GCA | GUA | 2 | A | V |
| *ycf*4 | 59010 | + | CUU | UUU | 1 | L | F |
| *cem*A | 60030 | + | CUU | UUU | 1 | L | F |
| *rpl*33 | 66272 | + | GCG | GUC | 2 | A | V |
|  | 66284 | + | GCG | GUG | 2 | A | V |
| *rps*18 | 66641* | + | UCC | UUC | 2 | S | F |
|  | 66901 | + | CUU | UUU | 1 | L | F |
| *psb*H | 73307 | + | GCA | GUA | 2 | A | V |
| *rpl*14 | 79537* | - | CGU | UGU | 1 | R | C |
| *rpl*22 | 82349* | - | UCA | UUA | 2 | S | L |
|  | 82008 | - | CCU | UCU | 1 | P | S |
| *rpl*23 | 148376* | + | UCU | UUU | 2 | S | F |
|  | 148394* | + | UCA | UUA | 2 | S | L |
| *ycf*2 | 147379 | - | GCC | GUC | 2 | A | V |
|  | 146558 | - | CUU | UUU | 1 | L | F |
|  | 146168 | - | CAC | UCA | 1 | H | Y |
|  | 146071* | - | UCU | UUU | 2 | S | F |
|  | 145649 | - | CAU | UAU | 1 | H | Y |
|  | 145519* | - | UCC | UUC | 2 | S | F |
|  | 144437 | - | CCA | UCA | 1 | P | S |
|  | 143708 | - | CCA | UCA | 1 | P | S |
|  | 143285 | - | CCC | UCC | 1 | P | S |
|  | 142486 | - | GCC | GUC | 2 | A | V |
|  | 142142 | - | CCC | UCC | 1 | P | S |
|  | 141958* | - | UCA | UUA | 2 | S | L |
|  | 141604 | - | CCU | CUU | 2 | P | L |
|  | 141562 | - | CCC | CUC | 2 | P | L |
| *rpl*32 | 110796 | + | CUU | UUU | 1 | L | F |
|  | 110823 | + | CAA | UAA | 1 | Q | STOP |
| *nhd*E | 114865 | - | CCG | CUG | 2 | P | L |
| *ndh*I | 116124* | - | UCA | UUA | 2 | S | L |
| *ndh*H | 119810 | + | GCU | GUU | 2 | A | V |
|  | 119243 | + | ACC | AUC | 2 | T | I |
| *rps*15 | 120381* | - | UCA | UUA | 2 | S | L |
| *ycf*1 | 124641 | - | GCU | GUU | 2 | A | V |
|  | 124540 | - | CAU | UAU | 1 | H | Y |
|  | 124420 | - | CUU | UUU | 1 | L | F |
|  | 124194 | - | ACU | AUU | 2 | T | I |
|  | 124182 | - | ACU | AUU | 2 | T | I |
|  | 124116 | - | GCA | GUA | 2 | A | V |
|  | 123885 | - | GCA | GUA | 1 | A | V |
|  | 123847  123846 | - | UCU | UUU | 1 and 2 | P | F |
|  | 123780 | - | ACA | AUA | 2 | T | I |
|  | 123186 | - | CCA | CUA | 2 | P | L |
|  | 122145 | - | ACA | AUA | 2 | T | I |
|  | 122007 | - | CCC | CUC | 2 | P | L |
|  | 122002 | - | CUU | UUU | 1 | L | F |
|  | 121128* | - | UCA | UUA | 2 | S | L |
